# Identification of Sjögren’s disease–associated T cell receptor motifs through deep sequencing

**DOI:** 10.1172/jci.insight.188496

**Published:** 2025-12-22

**Authors:** Ananth Aditya Jupudi, Michelle L. Joachims, Christina Lawrence, Charmaine Lopez-Davis, Bhuwan Khatri, Astrid Rasmussen, Kiely Grundahl, R. Hal Scofield, Judith A. James, Joel M. Guthridge, Christopher J. Lessard, Linda F. Thompson, A. Darise Farris

**Affiliations:** 1 Arthritis and Clinical Immunology Program, Oklahoma Medical Research Foundation, Oklahoma City, Oklahoma, USA.; 2 Department of Microbiology and Immunology, University of Oklahoma Health Sciences Center, Oklahoma City, Oklahoma, USA.; 3 Genes and Human Disease Program, Oklahoma Medical Research Foundation, Oklahoma City, Oklahoma, USA.; 4 Department of Medicine, University of Oklahoma Health Sciences Center, Oklahoma City, Oklahoma, USA.; 5 Department of Veteran’s Affairs Medical Center, Oklahoma City, Oklahoma, USA.

**Keywords:** Autoimmunity, Immunology, Autoimmune diseases, T cell receptor

## Abstract

CD4^+^ T cells predominate lymphocytic foci found in the salivary glands (SGs) of Sjögren’s disease (SjD) cases. Yet little is known about T cell receptor (TCR) repertoire features that distinguish cases from healthy controls (HCs), the relationship between SG and peripheral blood (PB) repertoires of cases, and antigens recognized by pathogenic T cell clones. We performed deep sequencing of bulk-sorted CD4^+^CD45RA^–^ PB T cells from SjD cases and matched HCs, and single-cell TCR sequencing of the same T cell population from labial SG biopsies of these cases. We found that clonally expanded SG CD4^+^ T cells expressed complementarity-determining region 3 (CDR3) sequences that were also detected in multiple copies in the blood of the same individuals with SjD. SjD cases displayed a “private” and restricted PB TCR repertoire with reduced clonotype diversity. We identified SjD-associated TCR motifs with the same putative antigen specificity shared between SGs and PB of cases. Their abundances in PB correlated with reduced salivary flow, linking these T cells with pathogenic disease features. Finally, we discovered 2 Ro60 epitopes eliciting an HLA-restricted immune response from expanded SG T cell clones. The comprehensive characterization of SjD TCR repertoires enables the discovery of target antigens and therapeutic strategies.

## Abstract 2

A comprehensive characterization of T-cell receptor (TCR) repertoire features in Sjögren’s disease patients, resulting in the discovery of disease-associated TCR motifs and canonical specificities.

## Introduction

Sjögren’s disease (SjD) is a chronic, rheumatic autoimmune disorder characterized by autoantibodies against Ro and focal lymphocytic infiltration of lacrimal and salivary glands (SGs) resulting in ocular and oral dryness, respectively (1). Systemic manifestations, including arthralgia, fatigue, and cutaneous and respiratory symptoms may also be observed, and mortality is mainly attributed to B cell lymphoma (2). SjD can occur in isolation or with other systemic autoimmune diseases, most commonly rheumatoid arthritis (RA) (3), systemic lupus erythematosus (SLE) (4), or systemic sclerosis (SSc) (5). SjD has a mean age of diagnosis in the 50s (6) and a highly skewed female/male ratio estimated at 14:1 (7).

Sicca and nonspecific symptoms mimicking other diseases often lead to SjD being misdiagnosed or undiagnosed (8). Etiology of the disease is unknown, although several studies implicate the role of multiple environmental factors and infectious agents in triggering and driving disease development (9). The DRB1*03:01/DQB1*02:01 (HLA-DR3/DQ2) haplotype is a genetic risk factor for SjD (10). Professional antigen-presenting cells (APCs) or glandular epithelial cells are hypothesized to present autoantigens to activated T cells, resulting in autoimmunity among persons with genetic predisposition (1, 11). CD4^+^ T cells are the predominant T cell type in the SG lymphocytic foci, contributing to germinal center–like structures and exhibiting a T follicular helper transcriptional profile (12). The antigens recognized by infiltrating SG CD4^+^ T cells are unknown, contributing to our lack of understanding of SjD pathogenesis.

Functional αβ T cell receptors (TCRs) expressed on the surface of T cells recognize antigenic peptides presented by MHC heterodimers on APCs, resulting in T cell activation, downstream signaling, and immune responses. The third complementarity-determining region (CDR3) is the most variable part of the TCR that makes direct contact with antigenic peptides and defines the antigen specificity of TCRs, while CDRs 1 and 2 stabilize the complex formed between the TCR and peptide-bound MHC (pMHC) (13). Analysis of CDR3 sequences and the TCR repertoire is therefore critical in understanding T cell–mediated autoimmunity.

Several studies have explored CDR3 sharing between individuals, biased usage of TCR variable (Vαβ) chain genes, conserved CDR3 motifs (14, 15), and the presence of peripheral and organ-specific T cell clonal expansions in autoimmune diseases, including celiac disease (15), RA (16), and SLE (17). While valuable in reporting early insights into conserved Vβ gene usage, prior TCR studies in SjD were limited by low-throughput techniques, small numbers of SjD cases, and the absence of specimens from healthy individuals to definitively elucidate SjD-associated TCRs (18–20). In our previous single-cell study, we observed SG CD4^+^ T cell clonal expansions in 9 of 10 SjD cases and demonstrated their association with tissue damage and oral dryness (14). We also observed CDR3 sequence similarities among multiple cases, suggesting shared recognition of antigens. However, our earlier study did not deeply sample the peripheral blood (PB), integrate the SG and PB TCR repertoires of SjD cases, or compare the PB TCR repertoires of SjD cases and healthy control individuals (HCs), another valuable strategy for identifying disease-associated CDR3 sequences. More recent studies characterizing TCRs in SjD through single-cell and bulk sequencing approaches have focused only on PB, excluding the glandular repertoire (21, 22). Among these, one study attempted to identify disease-associated PB-TCR clonotypes with a shallow bulk-sequencing dataset of 2.5–3 × 10^4^ or fewer unique clonotypes sampled across 3 time points from 260 SjD cases (averaging only 112 clonotypes per case) compared with publicly available TCRs from unmatched HCs not screened for autoimmunity or other exclusion criteria (21). Another single-cell study reported a few differences in broad diversity metrics and clonality of T cell subtypes between diseased and healthy repertoires but provided no insight into CDR3 sequence similarities or clonally expanded motifs among patients (22). A more comprehensive and systematic approach is needed to evaluate both glandular and peripheral TCRs from patients and utilize comparable data from matched HCs to identify disease-associated motifs in SjD.

In this study, we directly address whether CD4^+^ T cell clones that are expanded in the SGs of SjD cases are also detected and overrepresented in their blood. We investigate whether potentially pathogenic T cells from multiple SjD cases are likely to share similar antigen specificities by evaluating their TCR repertoires using GLIPH2 (23). We address key limitations of our previous analysis of the SjD TCR repertoire by conducting deep TCR profiling in PB and including demographically matched, verified HCs carrying the SjD-associated HLA class II risk locus. As both ethnicity and HLA haplotypes influence antigen presentation and the CDR3 sequences that can recognize a given antigen, careful case/control matching is critical for a valid study. By integrating SG and PB TCR data, we were able to identify disease-associated TCR motifs that are exclusively detected or preferentially enriched in the blood of SjD cases versus matched HCs and determine their association with clinical features of SjD. Our final goal is to identify disease-driving autoantigens. We utilized an in vitro screening approach to determine whether SjD-associated TCRs from expanded SG T cell clones recognize any portion of the canonical SjD autoantigens, Ro and La (24). To the best of our knowledge, we generated the largest collection of unique PB TCRs in SjD cases to date, averaging 6.92 × 10^4^ unique clonotypes per case, which when combined with SjD SG TCRs and compared to PB TCRs from HCs, enabled us to identify CDR3s associated with SjD. These are the first steps in developing PB-based strategies for the diagnosis of SjD as well as tools for following disease progression and response to experimental therapies.

## Results

### Clinical characteristics of participants.

SjD cases without overlapping SLE, RA, or SSc (*n* = 19), meeting the disease classification criteria recommended by the American College of Rheumatology (ACR) and the European Alliance of Associations for Rheumatology (EULAR) in 2016 (25), and an equal number of HCs matched for age, sex, and race were included in the study. All HCs had negative responses to the connective tissue disease screening questionnaire (26). All HCs and 16 of the 19 SjD cases carried the SjD-risk-associated haplotype alleles HLA-DR3/DQ2. The clinical and demographic characteristics of participants are listed in Table 1. No significant demographic or HLA differences were detected between the 2 groups. Nearly all (95%) SjD cases displayed focal lymphocytic sialadenitis in the labial minor SG (LSG) biopsy with a focus score of 1 or higher, and more than half (53%) had serum anti-Ro/La antibodies.

### Study design.

**Figure 1:**
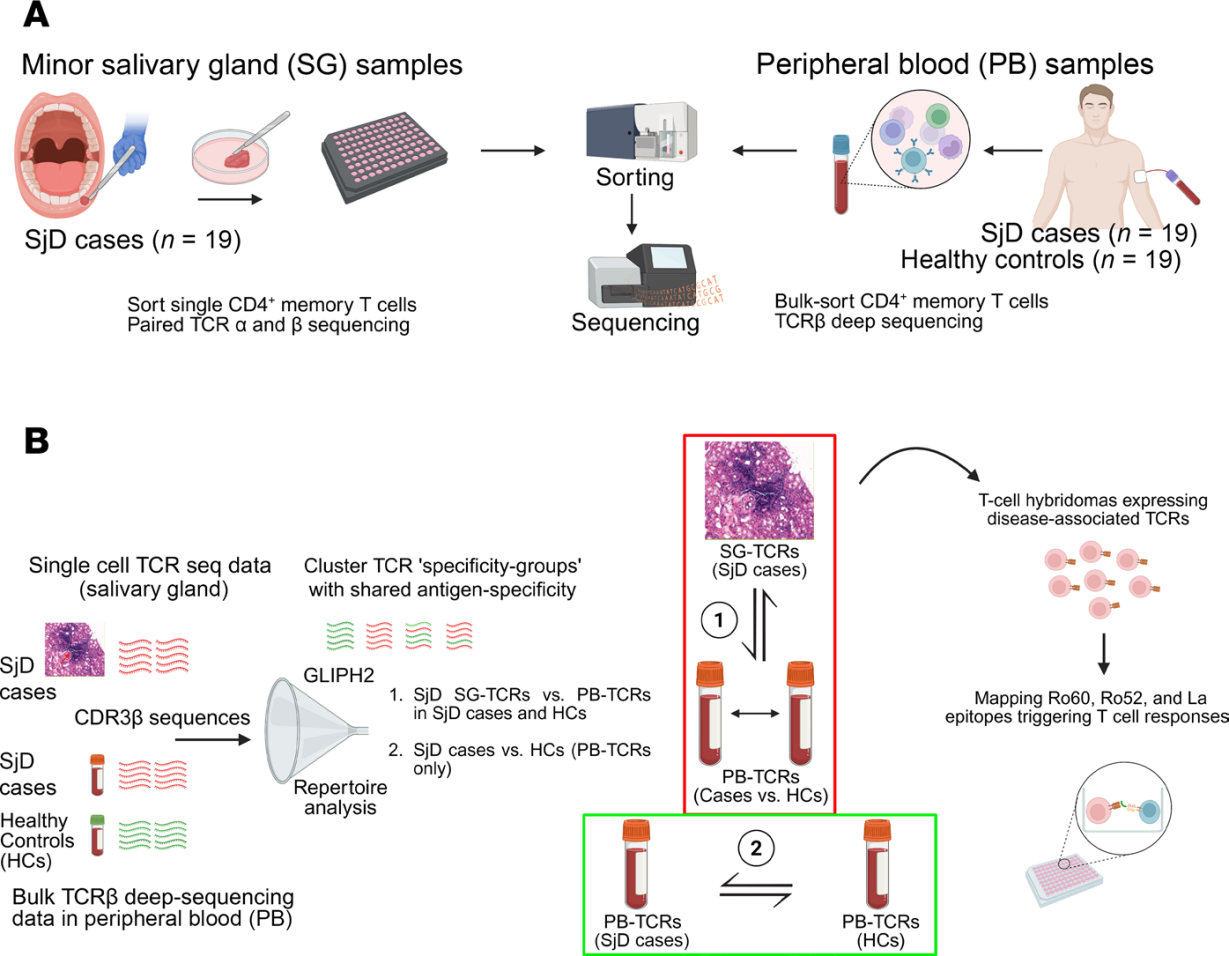
Workflow for the identification of SjD-associated TCRs. (**A**) Labial SG biopsies were collected only from SjD cases (*n* = 19), and PB was collected from both cases and HCs (*n* = 19). (**B**) TCR CDR3 sequences extracted from SG and PB were combined as input for antigen-specificity-based TCR clustering using GLIPH2. The resulting TCR clusters, in addition to individual CDR3 sequences in SGs and PB, were analyzed using 2 approaches: (a) The SG TCR repertoire of cases was compared with that of PB from both cases and HCs and (b) deep-sequenced CDR3β clonotypes found in the PB repertoires of cases and HCs were compared directly. TCRs of interest were tested for reactivity to overlapping peptides of the canonical SjD antigens Ro60, Ro52, and La.

Our overall goal is to identify TCRs involved in the pathogenesis of SjD with the ultimate aim of identifying antigens that trigger autoimmunity. Based on our previous work (14), we hypothesized that disease-associated SG T cells undergo clonal expansion due to an antigen-specific immune response. Therefore, we sorted single CD3^+^CD4^+^CD45RA^–^ memory T cells (Supplemental Figure 1A; supplemental material available online with this article; https://doi.org/10.1172/jci.insight.188496DS1) from biopsied LSGs of SjD cases and profiled their TCR repertoire, including documentation of clonally expanded TCRs. Paired TCRα and TCRβ sequences were obtained using multiplex TCR RT-PCR (*n* = 4) and single-cell RNA sequencing (*n* = 15). Our next question was whether SjD SG TCRs (expanded or not) could also be found in the PB, and whether these were unique to SjD cases or present at increased frequency compared with HCs. To address this, PB mononuclear cells (PBMCs) from the same SjD cases and matched HCs were bulk sorted for memory CD4^+^ T cells (Supplemental Figure 1B), and PB TCR deep sequencing was utilized to capture the circulating TCRβ repertoire (Figure 1A). A total of 1,693 unique CDR3 sequences (paired and unpaired TCRβ) were detected in SGs, while 1,123,116 CDR3β sequences were detected from the PB of SjD cases and 1,367,971 from the PB of HCs. The overlap between the SjD SG repertoire and both PB repertoires was analyzed by comparing the prevalence of SG TCRs found in the blood of cases versus HCs. Using the above 3 datasets, we identified “disease-associated” TCRs by (a) comparing the SG TCR repertoires of cases with the PB repertoires of cases versus HCs and (b) directly comparing the PB TCR repertoires of cases versus HCs (Figure 1B). TCR-clustering analysis, using GLIPH2 (23), was employed to identify groups of TCRs (i.e., motifs) that were likely to share antigen specificity. Then, the exclusive presence or differential abundance of these motifs in the PB of cases compared with HCs was used to identify disease-associated TCR motifs.

### SjD cases contain clonally expanded memory CD4^+^ cells in SGs and T cells with identical CDR3s in PB occurring at higher frequency in SjD cases than HCs.

**Figure 2:**
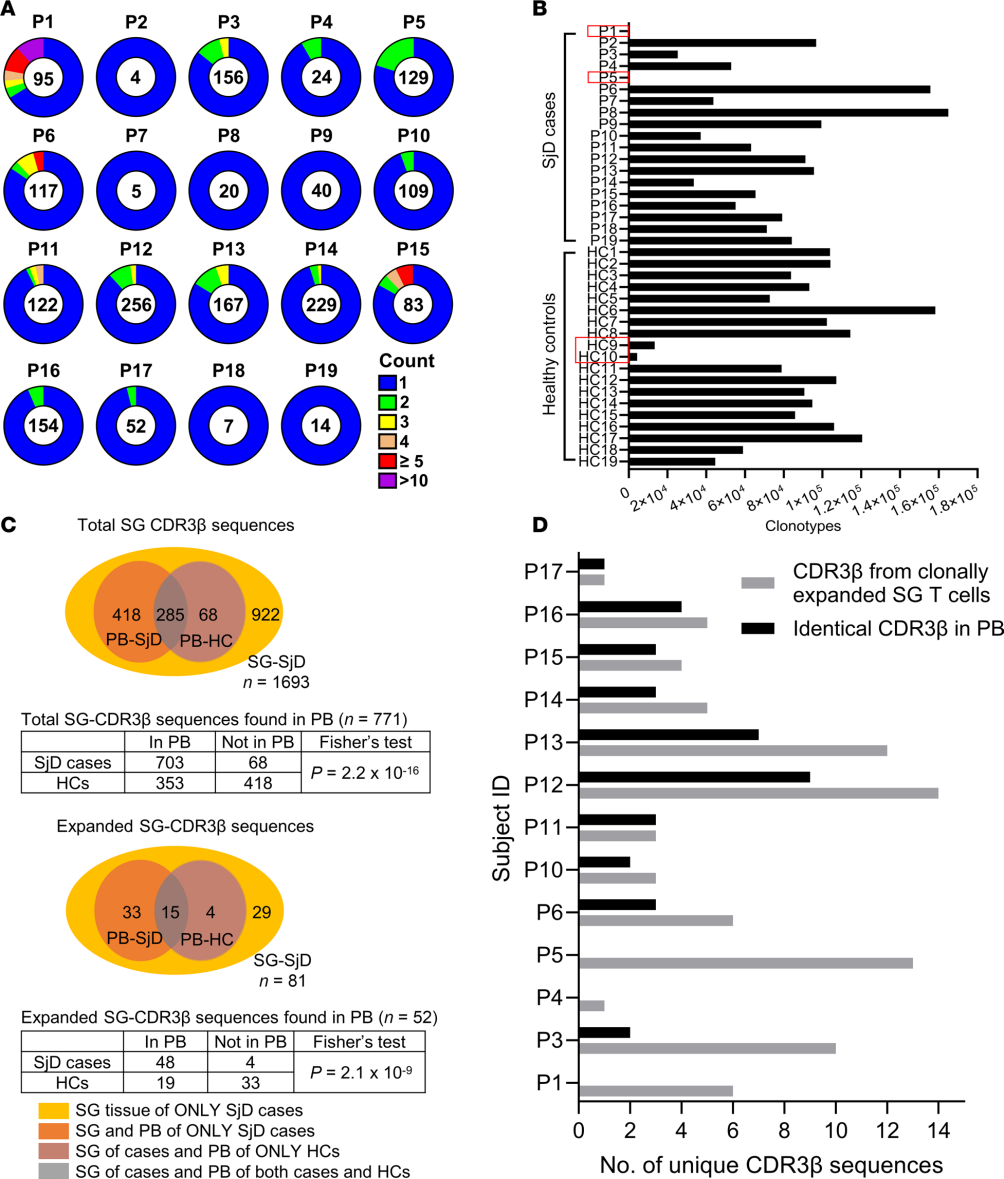
SG repertoire analysis reveals preferential sharing of clonal expansions and CDR3β sequences between the SGs and PB of SjD cases versus HCs. (**A**) Doughnut charts display the total number of TCRβ^+^ SG cells recovered inside the doughnut hole and the degree of clonal expansion (≥2 identical CDR3 nucleotide sequences) as shown by the color scheme. (**B**) Number of PB CDR3β clonotypes detected in all SjD cases and HCs are shown, with 4 individuals having lower numbers of clonotypes (red boxes). (**C**) Stacked Venn diagrams of the distribution of CDR3β sequences detected in total (top) and clonally expanded (bottom) SG T cells found in the blood of all SjD cases compared to HCs (2-sided Fisher’s exact test). (**D**) Number of clonotypes that are part of a clonal expansion in SG T cells and are also found in multiple copies in the PB of the same SjD case (*n* = 13).

Despite the relatively low number of SG TCR^+^ cells recovered from SjD cases (median = 95, range: 4–256), clonal expansions, defined as 2 or more T cells expressing nucleotide-identical CDR3β sequences or clonotypes in an individual, were detected in 13 of 19 SjD cases (Figure 2A). The number of cells constituting a clonal expansion represented a median of 11.7% (range: 3.8%–33.7%) of the total SG T cell repertoire in these 13 cases. The true incidence of clonally expanded T cells in SG of SjD cases is likely higher, as all cases with undetected SG clonal expansions had SG cell sample sizes of 40 cells or less (median = 14, range: 4–40). SG samples below this threshold likely underestimate clonal expansions and may skew low-incidence data. Much like the SG repertoire, the number of PB TCR clonotypes identified also varied considerably across different individuals, with 4 (2 cases and 2 HCs) yielding few productive clonotypes (Figure 2B).

Of 1,693 unique SG CDR3β sequences from SjD cases (*n* = 19), 922 (54.5%) were detected exclusively in SG, whereas 771 (45.5%) were identical to those also found in the PB repertoires of either cases, HCs, or both (Figure 2C). A majority of the latter (*n* = 771) were detected exclusively in the blood of SjD cases (54.2%) compared with HCs (8.8%) or were commonly detected in both groups (37%). This indicated a higher degree of overlap between SjD SG TCRs and PB TCRs found in SjD cases compared with those in HCs (Fisher’s exact test, *P* = 2.2 × 10^–16^; Figure 2C). Similarly, of the 81 unique CDR3β sequences corresponding to clonally expanded SG T cells, 52 were detected in PB repertoires, with a higher likelihood of such expanded clonotypes being detected in the PB of SjD cases (63.5%) compared with HCs (7.7%) (Fisher’s exact test, *P* = 2.11 × 10^–9^; Figure 2C).

Next, we investigated whether expanded SG clonotypes were detected in the PB of the same individual. Interestingly, 9 of 13 SjD cases exhibiting SG clonal expansions also showed multiple copies (≥5 cDNA copies) of at least one matching CDR3β clonotype in the PB of the same individual (Figure 2D and Supplemental Table 1), indicating overrepresentation in PB. Lack of detection of expanded SG clonotypes in PB for 2 cases (P1 and P5) is likely attributable to inadequate PB sampling (see Figure 2B). Furthermore, the degree of repertoire sharing between the SGs and PB of SjD cases is likely underestimated, owing to low sample sizes of SG TCRs in 7 of the cases (P2, P4, P7, P8, P9, P18, and P19). Thus, there is a higher degree of TCR repertoire sharing between the SGs and PB of SjD cases than the PB of HCs. Furthermore, we conclude that TCRs on clonally expanded SG T cells are commonly present and overrepresented in PB of the same SjD cases.

### SjD SG TCR motifs predicted to recognize the same antigen are preferentially shared with PB TCRs found in cases versus HCs.

To better understand the TCR repertoires of SjD cases and HCs, we utilized network analyses based on predictive models of shared antigen recognition to cluster nonidentical TCRs into motifs that have common features and are highly likely to have similar antigen specificity (27). GLIPH2 (23) is one of several tools (28–30) used to group TCRs most likely to interact with the same antigen into clusters referred to here as “specificity groups.” TCRs are grouped by global or locally enriched motif-based similarity, modeled on high probability antigen-contact sites in the TCR CDR3β region (23, 30), critical for establishing antigen contact (31).

**Figure 3:**
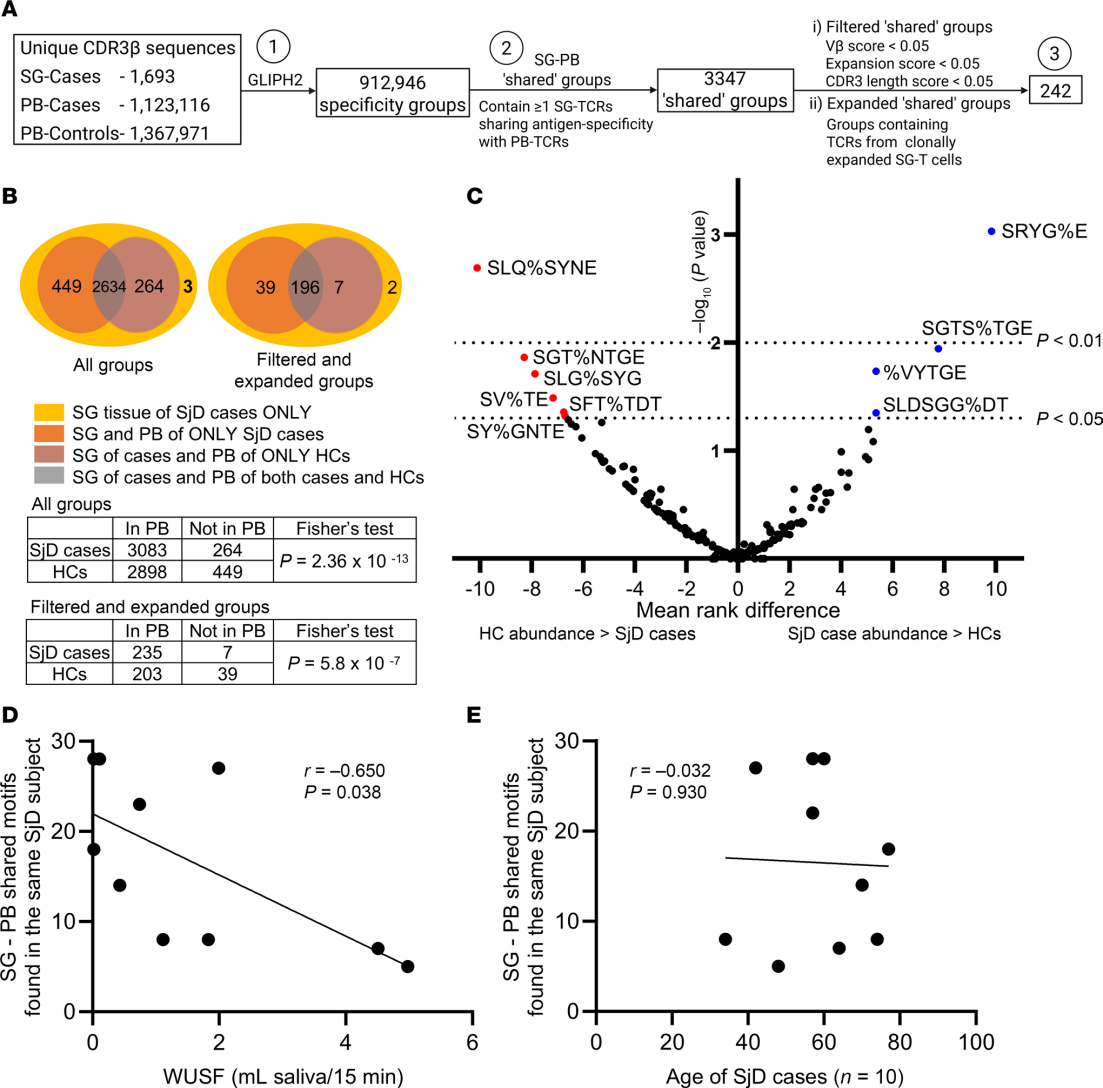
TCR motifs are commonly enriched in the PB and SGs of SjD cases. (**A**) Flowchart for the selection of antigen-specificity-based TCR clusters shared between SG and PB TCRs. (**B**) Stacked Venn diagrams show the number of TCR specificity groups detected in SGs only (yellow), SGs and PB of SjD cases only (orange), SGs of cases and PB of HCs only (brick red), or SGs of cases and PB of both cases and HCs (gray). Likelihood of SG TCR motif detection in the PB of cases compared to HCs was measured by 2-sided Fisher’s exact test. (**C**) Volcano plot comparing the PB abundances of CDR3β sequences constituting individual SG PB shared specificity groups, between SjD cases (*n* = 17) and HCs (*n* = 17) with adequate PB clonotype sampling (2-sided Mann-Whitney *U* test). TCR motifs significantly enriched among cases (blue) and HCs (red) were determined by *P* value (negative log_10_-transformed *P* values on *y* axis) and mean rank difference (*x* axis). (**D**) The number of SG PB shared motifs comprising glandular and circulating TCRs detected in the same SjD case among those with adequate sampling in both SGs and PB (*n* = 10) negatively correlates with whole unstimulated salivary flow (WUSF). (**E**) No correlation was seen between the number of motifs found in the SGs and PB of the same SjD individual and age. (**C**–**E**) Adequate sampling thresholds (per individual) for PB: >25,000 unique clonotypes; SG: >40 TCRβ^+^ cells. (**D** and **E**) Two-tailed Spearman’s rank correlation test.

All CDR3β sequences in the SGs (*n* = 1,693) and PB of SjD cases (*n* = 1,123,116) and PB of HCs (*n* = 1,367,971) were analyzed using GLIPH2, along with metadata on corresponding tissue of origin, gene usage, and frequency. This combined dataset generated a total of 912,946 specificity groups (Figure 3A), including 3,350 that contained at least one SG TCR. Of these, only 3 specificity groups were comprised exclusively of SG TCRs; the remaining (99.9%) contained both glandular and circulating TCRs, referred to here as SG PB “shared” specificity groups (*n* = 3,347). The number of shared groups containing CDR3β sequences detected exclusively in the SjD PB repertoire (*n* = 449, 13.4%) surpassed those found only in HCs (*n* = 264, 7.9%). Most groups (*n* = 2634, 78.7%) contained CDR3βs detected commonly in both cases and HCs (Figure 3B), as expected for antigenic exposures common to the human population. However, SG TCR-containing motifs were significantly more likely to be detected in the PB of cases when compared with HCs (Fisher’s exact test, *P* = 2.36 × 10^–13^; Figure 3B), indicating preferential sharing of antigen specificity between SjD SG and PB TCRs.

We identified 242 SG PB shared specificity groups exhibiting repertoire features of antigen selection and shared antigen specificity. These features include (a) specific filtering criteria such as enriched Vβ gene usage, CDR3β length conservation, and total CDR3β abundance enumerated as GLIPH2 scores (filtered shared groups; Supplemental Table 2) or (b) evidence of clonal expansion (expanded shared groups; Supplemental Table 3) within SG tissue (Figure 3A). Motifs displaying these features and found in the SGs were significantly more likely to be detected in the PB of SjD cases compared with HCs (Fisher’s exact test, *P* = 5.8 × 10^–7^; Figure 3B). Of these, 39 motifs were detected exclusively in SjD cases and were thus inferred as being disease associated. To identify SG PB shared specificity groups detected in the PB of both cases and HCs (*n* = 2634) but preferentially enriched in cases, abundances of individual PB CDR3β sequences comprising such groups were compared between cases and HCs. Here, abundance refers to the number of distinct cDNA molecules encoding a specific CDR3β. Four motifs among the filtered and expanded groups that are most likely to be antigen driven and share similar specificity occurred at a significantly higher abundance in the PB of SjD cases compared with HCs and were thus identified as a second subset of disease-associated motifs (Figure 3C and Supplemental Table 4), whereas motifs more enriched in HCs were also observed. An additional 9 motifs that were not among the filtered and expanded specificity groups were also enriched in SjD cases compared with matched HCs (Supplemental Figure 2A and Supplemental Table 4). In summary, we identified SjD-associated SG TCR motifs that occur exclusively or more abundantly in the PB of SjD cases compared with matched HCs.

### The prevalence of TCR motifs with shared antigen specificity detected in the PB and SGs of the same SjD case correlates with disease features.

All TCRs comprising SG PB shared specificity groups had CDR3β amino acid sequences of identical length; variations in sequences occurred at the same position, with amino acid substitutions of similar biochemical properties. In motifs such as S%WAGRPTDT (where % is a wildcard amino acid substitution) and KGLAGEYYE, nucleotide-identical TCR CDR3β sequences from clonally expanded SG T cells were detected in multiple copies in the PB of the same SjD case (Supplemental Figure 2B and Table 2). Some TCRs with dissimilar CDR3β nucleotide sequences still produced identical amino acid sequences by convergent recombination. Both germline and nontemplated nucleotides adding to junctional diversity during VDJ recombination contributed to this phenomenon, providing further evidence of antigenic selection. Antigen specificity was predicted to be shared between TCRs detected in the PB and SGs of the same individual in all 10 SjD cases with adequate sampling in both tissues (Supplemental Figure 2C). Strikingly, among these cases, the number of shared motifs correlated inversely with whole unstimulated salivary flow (WUSF) (Figure 3D) and was not driven by increasing age (Figure 3E). We then asked whether the PB abundances of shared motifs found in 5 or more SjD cases and HCs but preferentially enriched in cases (*n* = 13; Figure 3B) correlated with SjD clinical features (Supplemental Figure 3A). Increased PB abundance of the SST%GNT motif was positively correlated with disease activity as measured by the EULAR Sjӧgren’s syndrome disease activity index (ESSDAI) (32) and total weighted score of the ACR-EULAR disease classification criteria (Supplemental Figure 3B), suggesting a “pathogenic effect.” In contrast, abundance of the SGTS%TGE and S%AGPSYE motifs was associated with decreasing levels of serum IgG and decrease in total ESSDAI score, respectively, suggesting a “protective’ effect” (Supplemental Figure 3C).

### The PB TCR repertoire of SjD cases is more restricted, less diverse, and has more clonotypes with increased abundance compared with HCs.

**Figure 4:**
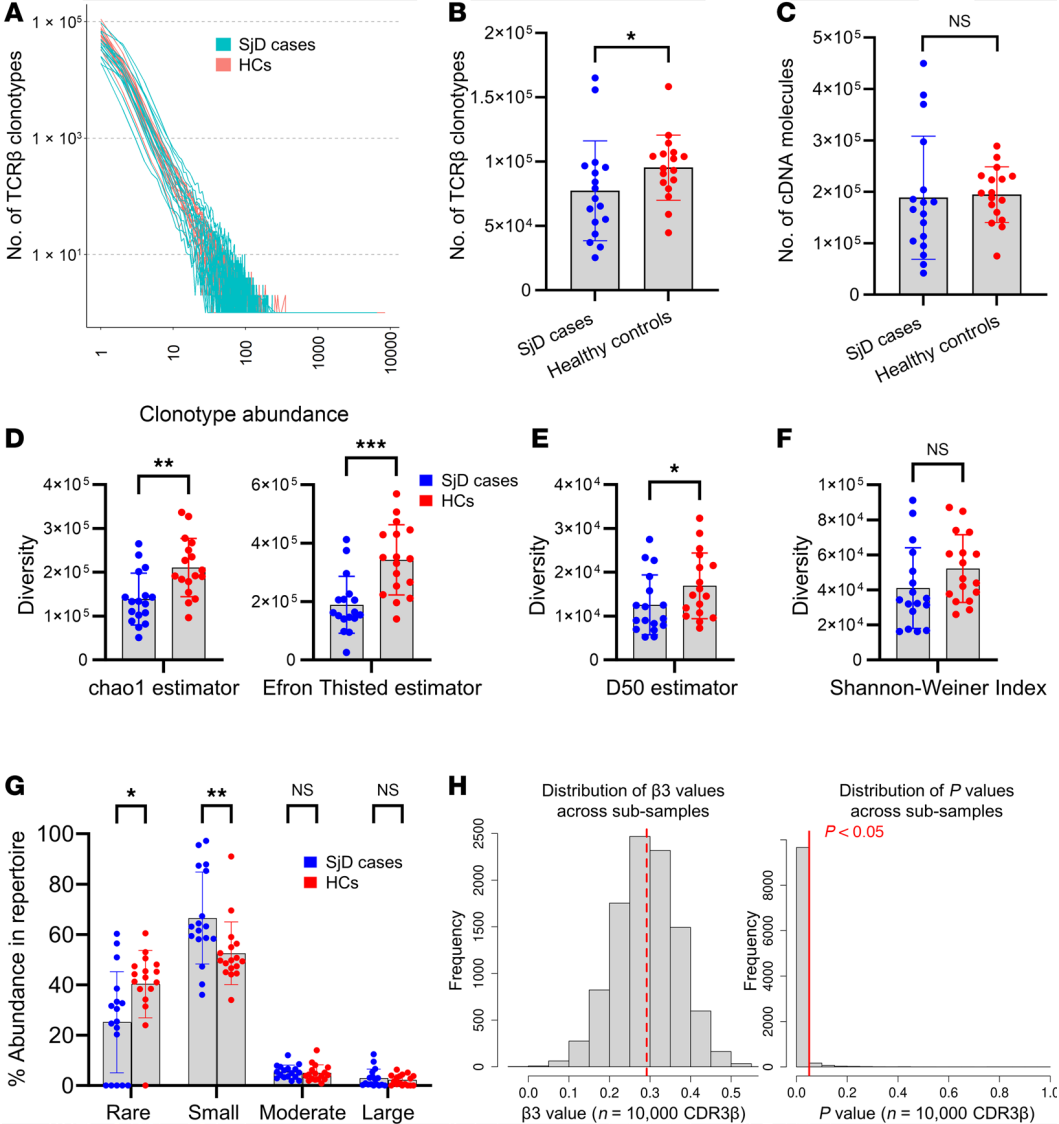
PB TCRs of SjD cases display reduced diversity, clonotypes with higher abundance, and a relatively private repertoire compared with HCs. (**A**) Distribution of TCRβ clonotype abundance across SjD cases and HCs. (**B**) Number of TCRβ clonotypes detected in the PB of SjD cases (blue) and HCs (red). (**C**) Number of TCR-encoding cDNA molecules detected in cases (blue) and HCs (red). (**D**–**F**) Comparison of TCRβ diversity between cases (blue) and HCs (red) measured by (**D**) chao1 estimator (species richness) and Efron-Thisted method, (**E**) D50 diversity index, and (**F**) Shannon-Weiner diversity index. (**G**) Proportion of repertoire space occupied by PB TCRs of rare (0 < *x* < 0.001%), small (0.001% < *x* < 0.025%), moderate (0.025% < *x* < 0.25%), and large (*x* > 0.25%) clonal abundance groups in SjD cases and HCs. (**H**) Distribution of effect sizes (β3, left) and corresponding *P* values (right), of the estimated change in pGen of CDR3β clonotypes against increase in their mean abundances, between SjD and HC repertoires (interaction term). Distributions are shown for 10,000 different subsamples comprising 10,000 CDR3β sequences each, from both cases and HCs. Positive β3 values (99.98%) reflect increase in the pGen of HC clonotypes over that of cases, with increasing mean clonotype abundance. Red dashed line (left) shows mean β3 across all subsamples (0.29). Red solid line (right) indicates iterations with *P* < 0.05 (96.62%). (**B**–**G**) Two-sided Mann-Whitney *U* test; data shown as mean ± SD. All comparisons were evaluated between cases (*n* = 17) and HCs (*n* = 17) with adequate PB TCR sampling (>25,000 unique clonotypes). *P < 0.05, **P < 0.01, ***P < 0.001. NS, not significant.

We next compared the total PB TCR repertoire between cases and HCs, including CDR3β sequences that were not shared with SGs. The input material used for cDNA library preparation (Supplemental Figure 4A), TCR sequence reads and depth (Supplemental Table 5), and overall clonotype abundance distributions (Figure 4A) of the PB TCRβ repertoires of SjD cases and HCs were similar. A significant reduction in the total number of unique TCRβ clonotypes was detected among SjD cases, suggesting restricted TCR repertoires with potential for increased occurrence of high-abundance clones (Figure 4B). This observation was not driven by differences in the sheer number of VDJ-mapped TCRβ cDNA molecules sampled, as these were similar in cases and HCs (Figure 4C). Furthermore, it was also not due to the number of T cells sampled on average in SjD cases versus HCs, as evidenced by the lack of correlation between cell and clonotype counts (Supplemental Figure 4B). We evaluated whether the diversity of SjD PB CDR3β sequences is reduced, resulting in a restricted TCR repertoire with fewer PB clonotypes in SjD cases compared with HCs. To address differences in sample sizes, we first plotted rarefaction curves and extrapolated the numbers of clonotypes detected in all individuals to the largest sample size. We found that the resulting numbers of clonotypes in cases remained lower than in HCs (Supplemental Figure 4C). Our findings also indicate that total species richness (chao1 estimate) of CDR3β sequences and estimated clonal diversity, including that of unseen species (Efron-Thisted estimate), is significantly reduced in cases compared with HCs, indicating the presence of fewer unique CDR3 “species” and a restricted TCR repertoire (Figure 4D). The diversity 50 (D50) index, which calculates the number of unique dominant (abundant) clonotypes occupying 50% of the total repertoire space, was also significantly reduced in cases compared with HCs (Figure 4E), indicating the increased presence of clonal expansions, as inferred previously (33), in the blood of SjD cases. Albeit not significant (*P* = 0.09), the SjD repertoire also had a lower Shannon-Weiner diversity index compared with HCs (Figure 4F).

No major differences were observed in Vβ gene usage for the most frequently used genes. TRBV5-1 and TRBV20-1 were the most dominant Vβ segments among both groups. Frequencies of other genes, including TRBV18, TRBV5-6, and TRBV6-8, were higher in cases, while TRBV29-1 was preferentially used in HCs, albeit all of these genes were expressed at very low frequencies (Supplemental Figure 4D). No differences were observed in the usage of TRBJ genes (data not shown).

### SjD PB clonotypes are more private and likely expand in response to antigen-driven selection compared with HCs.

A hallmark of T cell activation and response to antigens is the presence of high-frequency clones that have undergone selective expansion. To compare the degree of such expansions in SjD cases versus HCs, we divided CDR3β clonotypes into 4 distinct groups (rare, small, moderate, and large) based on their relative abundances and calculated the total TCR repertoire space occupied by clonotypes of each group. The “rare” group, consisting mostly of TCR singlets, was significantly enriched in HC repertoires compared with cases (Figure 4G), whereas clonotypes with “small” expansions were significantly more abundant in the PB of cases compared with HCs. No differences were observed for the “moderate” and “large” expansion groups. Whether clonotypes with increased abundance are always a result of directed immune responses is unclear. Overrepresentation of some TCRs in the general population, i.e., “publicness,” has been attributed to bias in VDJ recombination events as opposed to convergent selection (34–36). Elhanati et al. devised a model to predict the degree of publicness, which hypothesizes that TCR sequences with a high probability of generation (pGen) are more likely to be abundant in unselected repertoires. Therefore, while high-pGen clonotypes likely occur by recombination bias, low pGen can be indicative of antigen-specific clonotype selection as shown in SARS-CoV-2–specific T cell clusters (34, 37).

We used optimized likelihood estimate of immunoglobulin amino acid sequences (OLGA) (38) to calculate the pGen of PB CDR3β sequences and the linear relationship between pGen and mean abundance of individual CDR3β sequences in cases and HCs (Supplemental Figure 4E). Next, we calculated the degree to which pGen values in the SjD repertoire differ from those in HCs with increasing clonotype abundances (interaction term). We found that the pGen of clonotypes detected across HC repertoires increased significantly with increasing abundance relative to those in cases, suggesting lower pGen and a more “private” profile of clonotypes in the latter (*P* < 2 × 10^–16^; generalized linear model). To ensure that this difference was not skewed by high sequencing depth and the large sample size of CDR3β sequences included in the linear regression model, we repeated this analysis by randomly sampling 10,000 CDR3β sequences each from cases and HCs for a total of 10,000 iterations. A significant increase in pGen with increasing CDR3β abundance was observed in the HC repertoire compared with SjD cases (*P* < 0.05, β3 > 0) in 96.6% of all subsamples (Figure 4H). Therefore, we conclude that SjD TCRβ clonotypes with increased abundance were likely generated and expanded in response to antigen-driven selection and not by recombination bias, when compared with clonotypes in the HC repertoires.

### Multiple TCRβ motifs not found in SGs are shared across the PB repertoires of SjD cases.

**Figure 5:**
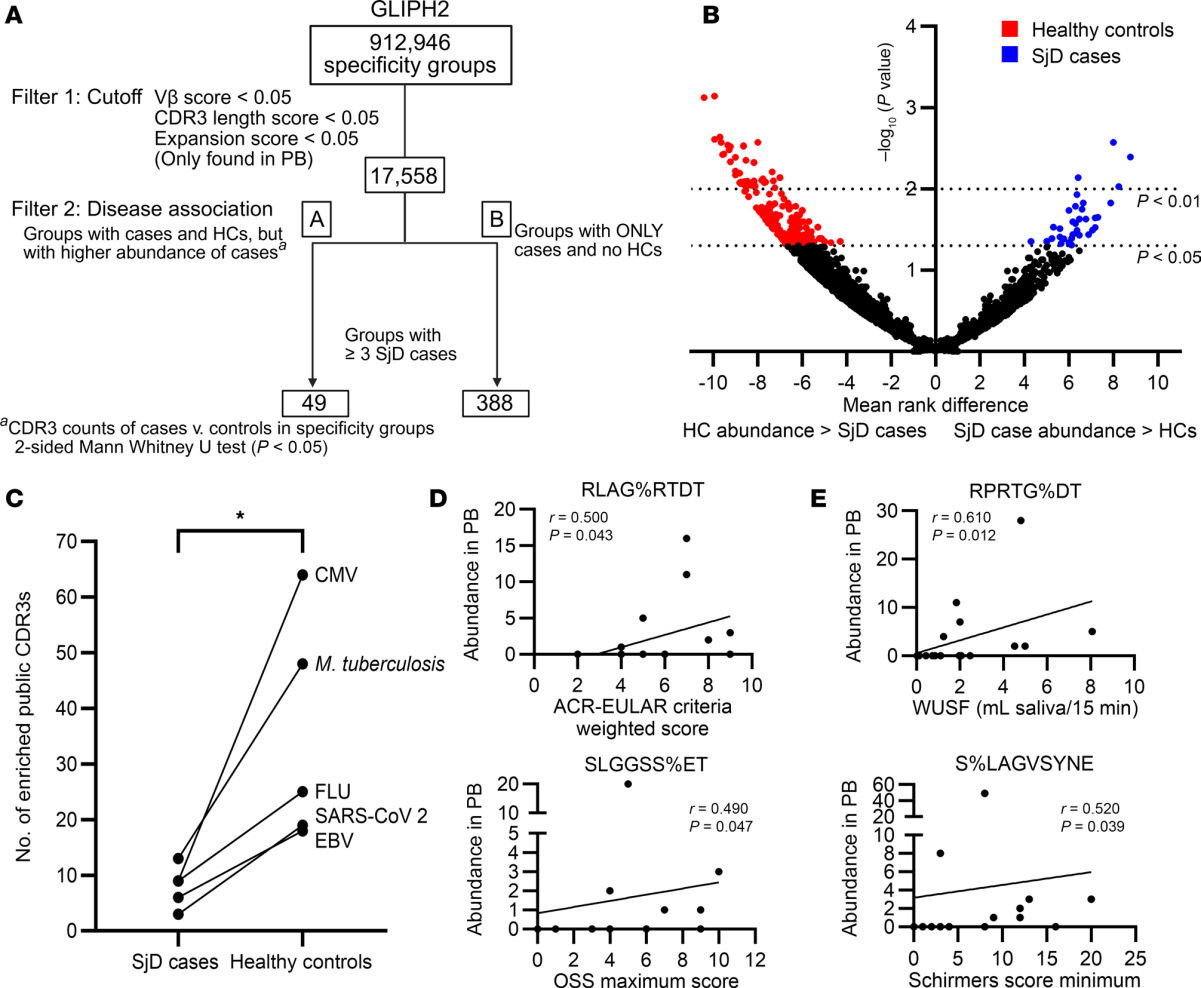
PB TCR repertoire of SjD cases contains disease-associated motifs not detected in SGs. (**A**) Flowchart for establishing antigen-specificity-based PB TCR clusters associated with SjD. Filter 1: All PB specificity groups were subjected to GLIPH2-based filtration criteria, including TRBV gene enrichment, CDR3 length conservation, and clonal expansion score. Filter 2: The abundances of resulting clusters in SjD and HC repertoires were compared to determine those exclusively present or preferentially enriched in 3 or more cases. (**B**) Volcano plot comparing the frequency of PB CDR3β sequences constituting specificity groups passing filter 1 cutoff, between cases and HCs (2-sided Mann-Whitney *U* test). Significantly enriched motifs among cases (blue; Supplemental Table 6) and HCs (red) were determined by *P* value (negative log_10_-transformed *P* values on *y* axis) and mean rank difference (*x* axis). (**C**) The number of known TCR specificities against select pathogens display significantly increased prevalence in HCs compared with cases (paired *t* test; *P < 0.05). (**D**) PB abundance of the motifs RLAG%RTDT (top) and SLGGSS%ET (bottom) correlates with worsening disease measures. (**E**) PB abundance of the motifs RPRTG%DT (top) and S%LAGVSYNE (bottom) correlates with improving disease measures. (**A**–**C**) All comparisons were evaluated between cases (*n* = 17) and HCs (*n* = 17) with adequate PB TCR sampling (>25,000 unique clonotypes). (**D** and **E**) Two-tailed Spearman’s rank correlation test.

To identify additional SjD-associated motifs not detected in the SGs, we applied the GLIPH2 filtration criteria (enriched Vβ gene usage, CDR3β length conservation, and total CDR3β abundance) to a total of 912,946 specificity groups and identified 17,558 groups comprising only PB TCRs (Figure 5A). We considered these groups as “disease-associated” if they were detected in (a) 3 or more SjD cases and any number of HCs, but with a significantly higher abundance in cases (*n* = 49; Supplemental Table 6 and Figure 5B) or (b) exclusively in 3 or more SjD cases (*n* = 388; Supplemental Table 7). All disease-associated specificity groups included at least one or more clonally overrepresented PB-TCR(s) (cDNA count ≥ 5) (Table 3). Convergent recombination was observed among more than one-fourth (26.5%) of all CDR3β amino acid sequences comprising disease-associated PB motifs, with the number of distinct nucleotide sequences encoding identical CDR3β aa sequences ranging between 2 and 16 (Supplemental Figure 5A). This convergence was not limited to germline nucleotides but included nontemplated nucleotides and occurred both within and across different SjD cases as seen in TCRs belonging to the SQE%TSSYNE and SPAVA%T motifs, respectively (Supplemental Figure 5B).

### Public TCR specificities are more prevalent in HCs compared with cases, suggesting an atypical immune system in SjD.

Notably, a large number of PB motifs that met the GLIPH2 filtration criteria were enriched in the PB of HCs compared with SjD cases (*P* < 0.05, Mann-Whitney *U* test; Figure 5B). We hypothesized that the preferential presence of TCRs with antiviral, “public” specificities in the general healthy population may have contributed to this occurrence. To investigate this, we conducted a query of all PB CDR3β sequences across 3 publicly available TCR databases (McPAS, VDJdb, and TBAdb) (39–41) and found that CDR3β clonotypes with specificities against pathogens such as CMV, *Mycobacterium* *tuberculosis*, influenza A, SARS-CoV-2, and EBV were 4 times more abundant in HCs compared with cases (Figure 5C). These findings are consistent with a less public SjD-associated PB TCR repertoire and suggest an atypical and potentially deficient immunological composition of the PB TCR repertoire in SjD cases.

### SjD-associated PB TCR motifs correlate with disease features in both protective and pathogenic ways.

To identify PB motifs associated with SjD clinical features, the abundances of disease-associated motifs shared among 5 or more SjD cases were correlated with objective features of disease (Supplemental Figure 6). Several motifs correlated positively with worsening disease measures, including weighted score of the ACR-EULAR criteria (RLAG%RTDT) and ocular staining score (OSS) (SLGGSS%ET) (Figure 5D). In contrast, the abundances of motifs like RPRTG%DT and S%LAGVSYNE were associated with improved disease features, including objective measures of oral (WUSF) and ocular (Schirmer’s score) dryness, respectively (Figure 5E). Some individual motifs were significantly associated with multiple clinical features, including both pathogenic (L%GRSYNE, SQVDLV%YNE, %DGGGTDT, and SLGGSS%ET) and protective (ARGQ%YE and SFGGRQ%T) ones (Supplemental Figure 6).

### TCRs from SG-expanded T cells of SjD cases recognize peptides derived from the canonical antigen Ro60.

**Figure 6:**
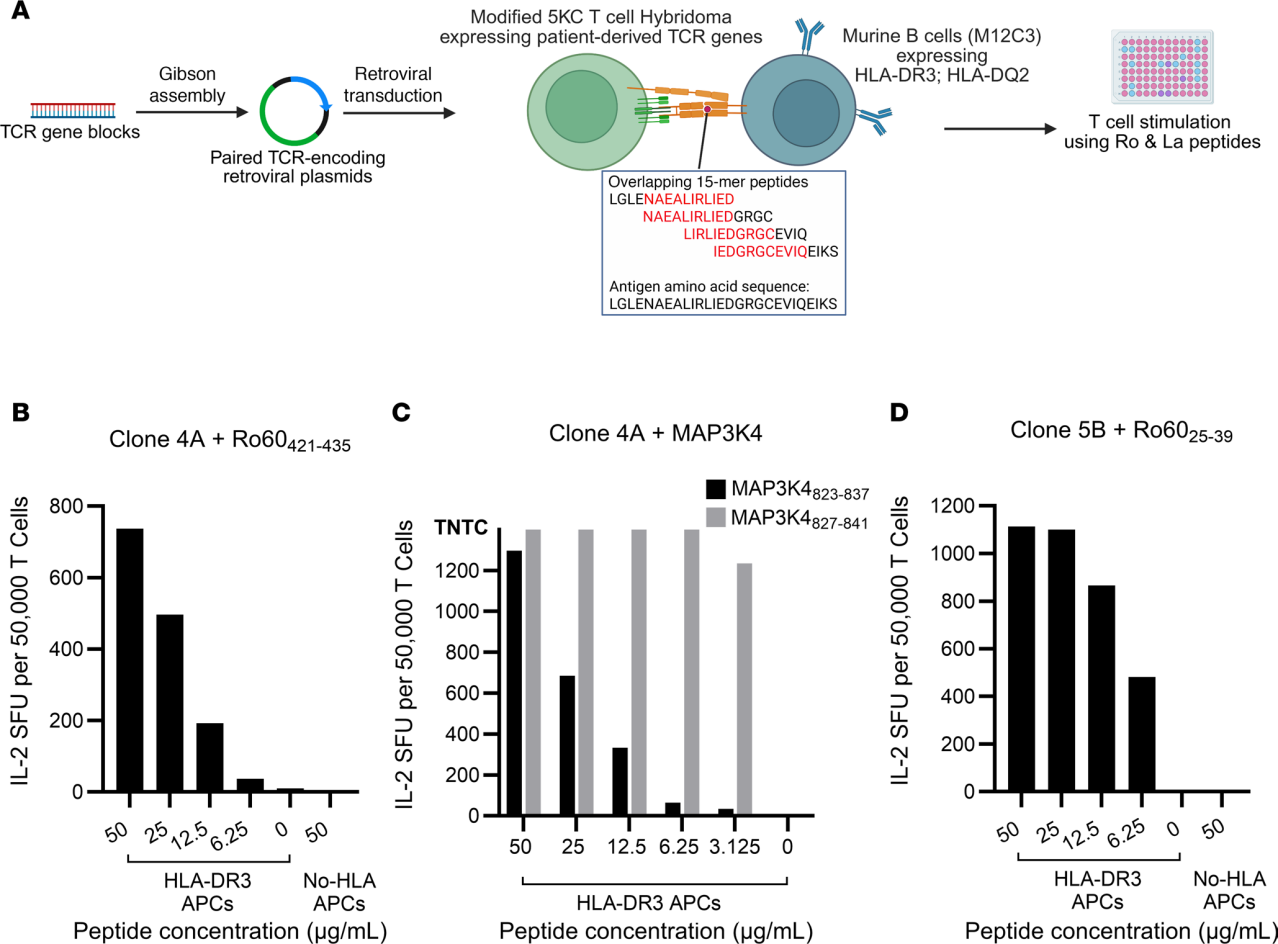
Discovery of Ro and La epitopes eliciting an immune response from select SjD-associated TCRs. (**A**) Stepwise process of generating TCR-expressing 5KC hybridoma T cells modified to express extra mouse CD3 genes, mutated human CD4 genes, and transduced with retroviral plasmids encoding patient-derived paired TCR genes. M12C3 B cell lines transfected with sequences encoding SjD risk–associated HLA heterodimers were used as APCs. 5KC-APC cocultures were incubated with 15-mer peptides (overlapping by 11 amino acids) spanning full-length Ro and La proteins. (**B**) IL-2 secretion (indicated as spot forming units [SFU]/50,000 T cells) by TCR 4A in response to decreasing concentrations of Ro60 (aa 421–435) in the presence of APCs expressing HLA-DR3 or no HLA alleles (negative control). (**C**) IL-2 secretion by TCR 4A in response to stimulation by decreasing concentrations of MAP3K4 aa 823–837 (black bars) and MAP3K4 aa 827–841 (gray bars) in the presence of APCs expressing HLA-DR3. (**D**) IL-2 secretion by TCR 5B in response to decreasing concentrations of the antigenic epitope Ro60 (aa 25–39) in the presence of APCs expressing HLA-DR3 or no HLA alleles (negative control). Number of SFU on higher concentrations of MAP3K4 peptides were too numerous to count (TNTC).

Next, we asked whether any TCRs from our study that were clonally expanded in SGs, overrepresented in the blood of cases, and included among disease-associated GLIPH2 motifs (*n* = 10), recognized epitopes from the canonical SjD antigens (Ro60, Ro52, or La) in a DR3- or DQ2-restricted fashion. In addition, we included TCRs from clonally expanded SG CD4^+^ T cells (*n* = 49) from 10 SjD cases that were previously published by our group (Supplemental Table 8). Engineered 5KC T cell hybridoma cell lines (42) were retrovirally transduced with plasmids encoding paired SG TCRs from SjD cases and cocultured with M12C3 B cell lines (43) transduced to express either HLA-DR3 or HLA-DQ2 on the cell surface. Overlapping 15-mer peptide libraries (11 amino acid overlap) spanning the length of Ro60, Ro52, and La (*n* = 365) were used to stimulate transduced T cells in cocultures, and epitopes eliciting dose-dependent T cell responses were identified through IL-2 ELISpot assays (Figure 6A).

In the first stage, involving 10 pools of 5 distinct T cell lines each, elevated IL-2 secretion was elicited by 9 distinct peptides (Supplemental Figure 7). Next, each of the 5 lines comprising a “positive” pool were individually cultured with varying concentrations of peptides that had elicited the positive responses. Using this approach, we identified a Ro60 epitope (aa 421–435) that stimulated dose-dependent secretion of IL-2 in T cells expressing TCR 4A restricted by HLA-DR3 (Figure 6B). The individual from whom this TCR was derived also tested positive for the presence of serum anti-Ro antibodies. Interestingly, this TCR was previously found to be specific for mitogen-activated protein kinase kinase kinase 4 (MAP3K4/MTK1) peptides presented by HLA-DR3 using the TScan-II platform for the identification of de novo CD4^+^ T cell epitopes (44). We verified this result using our screening approach and confirmed dose-dependent IL-2 secretion by TCR 4A in response to stimulation by 2 overlapping MAP3K4 15-mers (Figure 6C), indicating TCR cross-reactivity between Ro60 and MAP3K4. Although there was no obvious sequence similarity between the 2 epitopes, both fit the canonical HLA-DR3 binding motif, showing at least 2 anchor residues (Supplemental Figure 8). A second HLA-DR3–restricted Ro60 epitope (aa 25–39) elicited a similar dose-dependent response in cells expressing TCR 5B derived from an individual who had both serum anti-Ro and anti-La antibodies (Figure 6D). Transductants expressing TCRs 4A and 5B failed to secrete IL-2 when stimulated with Ro and La peptides in the presence of APCs lacking surface-expressed class II HLA, confirming HLA-DR3 restriction of the above 2 epitopes. No other epitopes were identified in these screening assays.

To validate natural processing and presentation of the 2 Ro60 epitopes, we screened TCR clones 4A and 5B for reactivity to full-length Ro60 presented by Flt3 ligand-induced dendritic cell–enriched (DC-enriched) splenocytes from an HLA-DR3 transgenic mouse. A dose-dependent secretion of IL-2 restricted by HLA-DR3 was observed for both T cell lines (Supplemental Figure 9, A and B). Additionally, reactivity of TCR clone 5B to peptides from full-length Ro60 presented by an available EBV-transformed B-lymphoblastoid cell line (B-LCL) autologous to the individual from whom the TCR originated was evaluated. In the presence of autologous IgG, enabling immune complex formation, clone 5B generated a dose-dependent IL-2 response to Ro60 that was attenuated by an anti–HLA-DR blocking antibody (Supplemental Figure 9C).

## Discussion

Autoimmunity in SjD could be triggered by a variety of antigens originating from an inflamed SG microenvironment, microbial or viral peptides, or even modified self-antigens (1). Incomplete knowledge of autoantigens involved in SjD diminishes our understanding of disease progression and has hampered the advancement of precise diagnostic approaches and effective targeted therapies (45). The contribution of T cells to SjD pathogenesis is well recognized, and a detailed case versus control study of the TCR repertoire holds the potential to uncover pathogenic clonotypes expressed on autoreactive T cells that can be harnessed for improved diagnosis or even TCR-based therapies. Therefore, we undertook a detailed analysis of TCR repertoires by evaluating antigen-experienced CD4^+^ T cells from the SGs and PB of SjD cases compared with PB repertoires of HCs. We present a systematic, targeted approach for identifying SjD-associated TCR motifs, discovered 2 Ro60 epitopes eliciting immune responses from SjD TCR-engineered T cells, and revealed several interesting repertoire features, incorporating (to our knowledge) the largest collection of SjD PB TCR clonotypes to date. This information, in addition to parallel studies detecting antigen specificities of autoantibodies in SjD, can lead to the identification of autoantigens triggering SjD.

The oral signs and symptoms of SjD reflect localized immunological activity mediated, at least in part, by SG-infiltrating lymphocytes, including CD4^+^ T cells (1). We found that all SjD cases sampled with more than 40 SG T cells contained clonal expansions occupying 4%–34% of the SG repertoire, indicating an immune response driven by antigen stimulation, as observed in pancreatic islets of patients with type 1 diabetes (T1D) (46).

The TCR repertoire of every individual is extensive, but only a few of the vastly diverse T cell clones that expand in response to infections, cancers, and autoimmunity (47) can be found in the PB. This diversity poses challenges in detecting pathogenic clonotypes circulating at low frequencies and warrants deep TCR profiling to reliably detect SjD clonotypes distinguishable from matched HCs. To address these challenges and identify clonotypes of interest, we sequenced PB TCRβ repertoires of individual cases (median: 19.6 × 10^6^ reads) and HCs (median: 20.2 × 10^6^ reads) beyond the range recommended for deep TCR repertoire profiling (48). We found multiple copies of PB TCRβ sequences identical to those expressed on the surface of clonally expanded SG T cells of all the same individuals, which to our knowledge is the first such observation in SjD. The PB TCRβ repertoires of SjD cases also comprised more clonotypes with increased abundances compared with HCs, consistent with a response to SjD antigen exposure.

Sharing of TCRs between target tissues and PB has been shown previously in autoimmune conditions like rheumatoid arthritis (RA) (49) and celiac disease (CD) (50) through antigenic stimulation of PBMCs (collagen peptides in RA) or antigen-specific tetramer-based T cell isolation (DQ2.5-glia-α2-tetramer in CD), respectively. In contrast, our study uncovered SG-PB TCR sharing without introducing biases in TCR repertoires such as stimulating the starting population or using tetramer-bound T cells that selectively bind high-affinity TCRs, approaches that often leave out a large proportion of those with low to moderate affinity known to play a bigger role in autoimmunity (47, 51).

The low-affinity binding interactions between the TCR and pMHC complex are mediated by a few residues from the antigenic peptide that accommodate “flexible” MHC-binding motifs and allow for different amino acid residues at primary MHC anchor positions (52). This binding degeneracy enables multiple TCRs to have specificity for the same antigen (53) and permits the detection of multiple antigens by a single TCR (54). We therefore utilized the GLIPH2 algorithm to look past TCR identity between SG and PB repertoires of cases, and to instead uncover SjD-associated TCR motifs based on likelihood of common antigen specificity. While a majority of SjD SG TCRs (55.1%) were distinct and not identical to those found in the PB of cases or HCs, very few of these shared putative antigen specificities with each other. A negligible fraction (0.09%) of GLIPH2 motifs were comprised of SG TCRs alone and the remaining were shared between SGs and PB. We demonstrated that SjD SG TCRs share putative antigen specificity preferentially with PB TCRs of cases compared with HCs. Notably, an increase in the number of motifs shared between SGs and PB of the same SjD case was associated with reduced salivary flow, a defining sign of SjD. These findings warrant further investigation of T cell migration between the 2 tissues. It is possible that TCRs with motifs found in both the SGs and PB play a crucial role in responding to antigens that trigger inflammatory responses, as recently demonstrated by T cells expressing a disease-associated CDR3β motif isolated from the blood and synovial fluid of individuals with ankylosing spondylitis and from the blood and eyes of those with acute anterior uveitis (55).

We also compared only the PB repertoires of cases to that of HCs to identify additional circulating disease-associated TCR motifs. We identified PB TCR motifs exclusively detected or selectively enriched in the PB of SjD cases versus HCs that were associated with SjD disease measures. However, most PB TCR motifs were shared between the repertoires of both cases and HCs. Fewer motifs were enriched exclusively within HCs, and an even smaller number only in cases. We hypothesize that sharing of these motifs between cases and HCs can be attributed to public TCRs of known viral or microbial specificities found in large proportions of the general population. A significantly higher incidence of such specificities in HC repertoires compared with those of SjD cases suggests an atypical or deficient nature of the latter. This finding is consistent with SjD cases having an increased frequency and burden of serious infections (pneumonia and sepsis) compared with those without SjD in longitudinal studies (56), as well as a heightened risk of hospitalization from mycobacterial, bronchopulmonary, and urinary tract infections (57), where deficient local immunity is a suspected mechanism (58).

We found that SjD PB TCR repertoires contained fewer clonotypes than HCs despite similar numbers of input cells, levels of input RNA, sequencing depth, and number of cDNAs detected between the 2 groups. PB TCR diversity across multiple indices was also significantly reduced among cases compared with HCs, suggesting restricted TCR usage that is most likely driven by antigen-mediated selection and expansion of T cells. Furthermore, pGen analysis revealed that clonotypes with increased abundance in cases are likely indicative of a more “private” SjD-specific repertoire compared with that in HCs and are not a result of VDJ recombination bias. Although we observed minor but detectable preferential usage of TRBV18, TRBV5-6, and TRBV6-8 in SjD PB TCR repertoires, we did not observe the same biases reported in other studies (Supplemental Table 9). Studies relying on shallow sampling are prone to detect inherent recombination biases, and those of low magnitude may occur by chance.

Our analysis expands the collective understanding of TCR repertoire characteristics in SjD, including motif sharing between SGs and PB, as well as the relationship between specific motifs and clinical manifestations of SjD. We leveraged systematic, statistical approaches to identify disease-associated TCRs, the specificity and pathogenic potential of which we aim to determine in future work. The canonical autoantigens Ro and La have long been associated with SjD (59), and autoantibodies against the same are known to be elevated among cases. The presence of serum anti-Ro antibodies is also routinely used for diagnosis and included in the 2016 ACR-EULAR SjD classification criteria. We therefore investigated whether any of our potentially pathogenic TCRs that are expanded in the SG, overrepresented in the blood, and are included in SjD-associated GLIPH2 motifs, recognize Ro and/or La epitopes.

Our discovery of 2 HLA-DR3–restricted Ro60 CD4^+^ T cell epitopes in the SG tissue of SjD cases implies a role for T cell help in generating SjD-associated anti-Ro60 autoantibodies. However, only 2 out of 50 SjD clones tested reacted with peptides from Ro and/or La. While the DR3/DQ2 haplotype is associated with autoantibodies against Ro and La (24, 60), recent genetic studies suggest that this association may be primarily driven by C4 gene copy number and not by the HLA loci (61). Thus, we cannot exclude the possibility that additional TCRs we screened may be specific for Ro and/or La epitopes but restricted by other class II heterodimers. Although the HLA scoring function of GLIPH2 can track the enrichment of specific HLA alleles (DR3 or otherwise) corresponding to SjD TCR motifs, this feature could not be utilized in our study due to a bias created by an overrepresentation of cases with HLA-DR3/DQ2 alleles. The specificity of the other disease-associated TCRs is an open question but may arise as a consequence of tissue damage and contribute to the chronicity of glandular involvement. Indeed, we recently discovered a number of new autoantibody specificities in both Ro-seropositive and -seronegative SjD cases that showed varied HLA associations and are part of pathways previously implicated in SjD (62).

Our finding that expanded SG clone 4A recognizes a Ro60 peptide in addition to a MAP3K4 epitope, as reported in our recent study featuring TScan-II (44), highlights the potential importance of TCR cross-reactivity in SjD. Using engineered mouse B cells as APCs, clone 4A exhibited an approximate 7-fold lower-affinity response to the Ro60_421–435_ epitope (calculated EC_50_ = 12.6 μM) relative to that of an epitope of MAP3K4 (calculated EC_50_ = 1.73 μM), which is expressed in SG ductal cells at higher levels in SjD cases compared with HCs. EC_50_ values were also calculated from experiments where full-length Ro60 was presented by the more physiologic primary DC–enriched splenocytes from an HLA-DR3–transgenic mouse. These values were 126 nM and 2.53 nM for clones 4A and 5B, respectively (Supplemental Figure 9), indicating that they are among the class of low-affinity, autoreactive TCRs previously reported in other autoimmune diseases including multiple sclerosis (MS) and T1D (63). Further studies uncovering the specificities of the SjD-associated TCRs presented by the full complement of patient HLA heterodimers will be needed to elucidate the relative importance of TCR specificity for canonical, SG tissue–expressed, or cross-reactive antigens in SjD.

A limitation of our study is that we did not track the abundance of SjD-associated clonotypes or motifs over the course of disease. However, our finding that SjD-associated SG T cell clones are also detected in PB means they can be tracked over time in the blood. Future efforts will be directed at uncovering the specificities of such SG T cell clones, further enabling pMHC multimer–based identification of pathogenic clones over time to track associations with disease activity and progression. Such studies could also be valuable in evaluating the efficacy of new therapeutic strategies. Secondly, our antigen screening approach was limited to measuring TCR reactivity in an engineered cell line (IL-2 secretion). Further experiments are needed to evaluate the secretion of other cytokines by primary patient T cells expressing SjD-associated TCRs.

In conclusion, we provide an extensive analysis of the TCR repertoire in SjD cases, incorporating TCR sequences from both SGs and PB, including the largest collection of the latter to date. We revealed notable insights into characteristics of the SjD TCR repertoire, including restricted TCR diversity, TCRs with higher abundance, and a relatively private repertoire among SjD cases compared with matched HCs. We demonstrated the presence of expanded SG clones in the PB of cases, discovered SG PB shared TCR motifs associated with systemic disease measures, and detected motifs shared between SGs and PB of the same individual associated with reduced salivary flow, necessitating further studies focusing on the interplay between the 2 tissues during the course of disease. The presence of potentially pathogenic or protective motifs occurring at higher abundances in the PB of cases compared with HCs underscores the peripheral component of SjD that may play a vital role in the phenotypic heterogeneity of the disease. Furthermore, our approach of determining the SjD-associated TCR specificity groups and subsequent identification of 2 Ro60 epitopes sets the stage for future longitudinal research and deeper exploration of SjD-associated antigen specificities, enhancing our understanding of SjD pathogenesis and informing potential targeted therapeutic strategies.

## Methods

Further information can be found in Supplemental Methods.

### Sex as a biological variable.

Although our study included both men and women, the rarity of SjD in men precluded an analysis of the data using sex as a biologic variable.

### Participant cohort and sample collection.

Participants attending the Oklahoma Sjögren’s Research clinic (OSRC) between October, 2011 and March, 2012 and between August, 2017 and February, 2020, meeting the 2016 ACR-EULAR research classification criteria for SjD (25), and lacking overlapping rheumatic disease diagnoses were considered for study inclusion. Individuals testing positive for HLA-DR3 and HLA-DQ2 alleles were given preference. Clinical examinations, objective assessment of ocular and oral dryness, LSG biopsies for single-cell sorting and focus scoring, and collection of PB samples and other pertinent clinical laboratory data were performed in the OSRC, as previously described (64). Serum autoantibodies were measured using Bioplex 2200 ANA screens. HCs matching the demographics (age, sex, and race) of SjD cases, testing positive for the presence of HLA-DR3 and -DQ2 alleles, and having negative responses to the Connective Tissue Disease Screening Questionnaire (CSQ) (26) were recruited through the Oklahoma Rheumatic Disease Research Cores Center. Class II HLA alleles for all individuals were imputed from genome-wide association study data using a next-generation genotype imputation server (65) (Supplemental Table 10).

### SG and PB processing and cell sorting.

Fresh LSG biopsies from SjD cases were processed for single-cell sorting as described previously (66). Full-length cDNA was amplified from single cells from previously described individuals, amplified for TCRα and TCRβ sequences by multiplex PCR, and analyzed by Sanger sequencing (14). Cells from additional individuals were processed using an adapted Smart-Seq2 method, as described by Eltahla et al. (67). Briefly, CD3^+^CD4^+^CD45RA^–^ cells were bulk sorted using a FACSAria Illu (BD Biosciences), then singly sorted into 96-well PCR plates containing 4 μL/well of catch buffer (RNAsin, 0.2% Triton X-100, 10 mM dNTPs, 5 μM oligo-dT30-VN) using a MoFlo XDP (Beckman Coulter). Plates were immediately sealed and stored at –80°C. Cells were then processed by plate to yield full-length cDNA (67), which was used to prepare sequencing libraries using the Nextera XT (Illumina) workflow. Single cell libraries were pooled, then sequenced with paired-end 150-bp reads to a target depth of 0.5 × 10^6^ reads/cell on an Illumina NovaSeq 6000. Density gradient centrifugation (Lympholyte-H, Cedarlane) was used to isolate PBMCs. PBMC suspensions were stained with the following anti-human antibodies: (a) Alexa Fluor 488 anti-CD8 (clone RPA-T8, BD Biosciences), (b) PE anti-CD3 (clone SK7, BioLegend), (c) PE-Cy5 anti-CD4 (clone RPA-T4, BioLegend), (d) V450 anti-CD45RA (clone HI100, BD Biosciences), and (e) BV605 anti-CD19 (clone HIB19, BioLegend), and bulk-sorted for CD45^+^CD3^+^CD4^+^CD45RA^–^CD8^–^CD19^–^ T cells with a target count of 0.5 million cells for each individual on a FACSAria (BD Biosciences), and stored in TRIzol at –80°C until use.

### RNA extraction and cDNA synthesis.

RNA was extracted and purified using RNeasy MinElute kits (QIAGEN) according to the manufacturer’s instructions. RNA yields were measured using the Qubit high-sensitivity assay kit (Invitrogen). A median of 400 ng of RNA (range: 70–400 ng) was used for cDNA synthesis (Supplemental Table 5). A modified cDNA-based library preparation protocol was adopted to prepare libraries suitable for TCR deep sequencing (48). First-strand cDNA was labeled with UMI barcodes using the SmartScribe RT kit (Takara Bio). The products were purified using Qiaquick PCR purification kits (QIAGEN). Two-stage amplification of TCRβ chains was performed as described by Egorov et al. (68). Samples with similar concentrations were pooled together for indexing and library preparation using NEBNext Ultra II DNA kits from Illumina (New England Biolabs). Adaptor-tagged DNA from pooled samples was subjected to size selection, and excess adaptors were removed using KAPA Pure beads (Roche Sequencing Solutions). Following a PCR amplification step using Illumina universal index primers, TCRβ libraries were gel purified, and their integrity verified using a TapeStation (Agilent Technologies).

### Generation of TCR transductants carrying SjD-derived TCR genes.

TCRs for retroviral expression were selected from expanded SG clonotypes (14) and disease-associated TCRs from SjD cases in the current study. Double-stranded DNA fragments (gBlocks, Integrated DNA Technologies) of full-length paired TCRα and TCRβ variable/CDR3 gene sequences and mouse TCRα constant region with 3′ self-cleaving 2A protease derived from porcine teschovirus-1 (69) were assembled into a murine stem cell virus–based (MSCV-based) vector through Gibson assembly (70). The MSCV vector also contained the mouse TCRβ constant region sequence and yellow fluorescent protein (YFP) genes (71). Plasmids were screened by PCR and Sanger sequenced to verify successful assembly of all TCR gBlocks. Plasmids were transfected into the retroviral packaging cell line Phoenix-Eco (ATCC, CRL-3214), producing a murine retrovirus that expressed a human-mouse (constant region) hybrid TCR. The cells for retroviral transduction were modified 5KC murine hybridoma T cell lines (42) lacking endogenous mouse TCR expression. These cells were engineered by retroviral transduction to have increased mouse CD3 expression and mutant human CD4 expression (amino acid substitutions: Q40Y and T45W) (72) to improve TCR-pMHC interaction and signaling in the murine 5KC cells (71). Briefly, these cells were spin-infected with retroviruses carrying paired TCR genes of interest from SjD cases in a 2-step process as described previously (73, 74). The resulting TCR transductants were cultured for 2 days, then sorted against vector-encoded YFP and ametrine markers on a MoFlo XDP (Beckman Coulter). The MSCV vector and 5KC murine hybridoma T cell lines were provided by Maki Nakayama (University of Colorado Anschutz, Aurora, Colorado, USA).

### Measurement of T cell responses to antigenic peptides by ELISpot.

Reactivity of the 5KC TCR transductants to antigenic peptides was measured by ELISpot assays using the Immunospot mouse-IL2 single-color ELISpot kit (CTL). Briefly, TCR-transduced 5KC cells (pool of 5 cell lines in first stage) were cocultured with HLA-DR3– and HLA-DQ2–transfected mouse M12 B-cell lines (43, 71), provided by Maki Nakayama, in the presence of Ro and La overlapping Pepsets at a final concentration of 50 μg/mL in duplicate wells on high-protein-binding 96-well PVDF filter plates coated with an IL-2 capture antibody (CTL), for 24 hours at 37°C and 5% CO_2_. Stimulation of TCR-expressing 5KC cells with hamster anti-mouse CD3ε antibody (clone 145-2C11, BD Biosciences) at 0.5 μg/mL (in duplicate) was used as a positive control. After coculture, ELISpot plates were developed with biotinylated detection antibody (CTL), followed by streptavidin alkaline phosphatase, and the enzymatic blue developer solution in the Immunospot kit. IL-2 production was measured by counting the number of spot-forming units (SFU) on ELISpot plates using an Immunospot analyzer (CTL). The mean of IL-2 SFU on duplicate wells was calculated, and a threshold for positive T cell responses was set at greater than 2 SD from the mean count of IL-2 SFU on negative control (zero peptide) wells. Results from “positive” pools were confirmed by peptide titration (singlet wells) using individual TCR-transduced 5KC cell lines. Please see Supplemental Methods for measurement of T cell responses to full-length Ro60 protein.

### Statistics.

Nonparametric tests (Spearman’s correlation and Mann-Whitney *U* test) were used where the data being compared did not follow a normal distribution, whereas parametric tests (Pearson’s correlation, paired *t* test, and unpaired *t* test with Welch’s correction) were used for the comparison of normally distributed data, with *P* less than 0.05 being considered significant. To maintain adequate sampling and minimize the introduction of type II errors, we established minimum sampling thresholds of more than 40 SG T cells and fewer than 25,000 PB clonotypes for statistical comparison of clonotype abundances or prevalence between individual cases and HCs as indicated in the figure legends. These data were analyzed using Prism 9.5.1 (GraphPad Software). Statistical models and plots for pGen analysis were generated using appropriate functions on R version 4.1.2 (link provided below in *Data availability*). Additional methods on deep sequencing, all computational analyses including GLIPH2, cell lines, and peptide libraries are included in Supplemental Methods.

### Study approval.

This study was approved by the Oklahoma Medical Research Foundation (OMRF) Institutional Review Board (protocols, IRB 18-07, IRB 07-12, and IRB 11-18). Participants gave written informed consent to participate in the study.

### Data availability.

PB TCRβ sequences were deposited in the NCBI Sequence Read Archive (SRA PRJNA1152703). TCR CDR3 sequences from targeted single-cell RT-PCR from 2 SjD cases that were previously reported (14) are available in GenBank (KX075774-KX075864, KX076847-KX077037). Sequences from 2 additional cases have been deposited in GenBank (PQ477904-PQ477919). TCR CDR3 sequences obtained from single-cell RNA sequencing data are found in Supplemental Table 11, and whole transcriptome reads are being submitted to dbGAP (phs002723.v1.p1) in association with a separate study. Custom code used for data extraction and analysis are available at https://github.com/OMRF-CBDS-FarrisD-Lab/SjD-TCR-Analysis Values for all data points in graphs are reported in the Supporting Data Values file.

## Author contributions

ADF conceived the study. AAJ, MLJ, and CL acquired the data. AAJ, MLJ, BK, LFT, and ADF analyzed the data. AR and RHS evaluated patients. KG managed clinical data. JAJ and JMG recruited and characterized healthy control individuals, and CLD processed their PB samples. ADF, CJL, and RHS contributed to patient recruitment and characterization. AAJ, MLJ, LFT, and ADF wrote the manuscript. All authors reviewed and edited the manuscript.

## Funding support

This work is the result of NIH funding, in whole or in part, and is subject to the NIH Public Access Policy. Through acceptance of this federal funding, the NIH has been given a right to make the work publicly available in PubMed Central.

- NIH grants R01AR074310 (to ADF), P50AR060804 (PI: Kathy L. Sivils; ADF, RHS, AR, and CJL), R01AR073855 (to CJL), U54GM104938 and UM1AI144292 (to JAJ), and P30AR073750 (to JAJ and JMG).
- Innovative Research Award from the Rheumatology Research Foundation (to ADF).

## Supplementary Material

Supplemental data

Supplemental tables 1-12

Supporting data values

## Figures and Tables

**Table 1:**
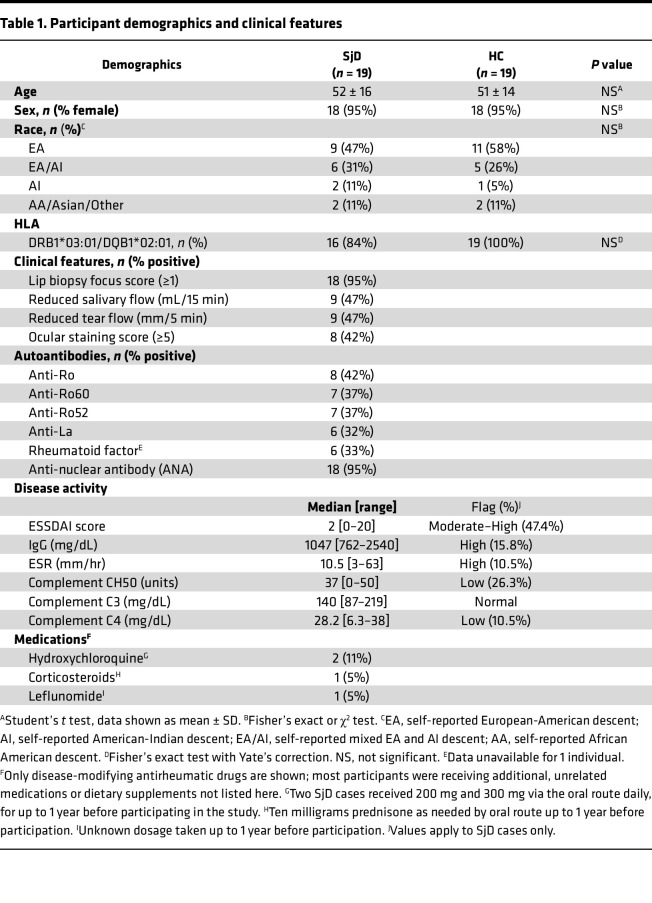
Participant demographics and clinical features

**Table 2:**
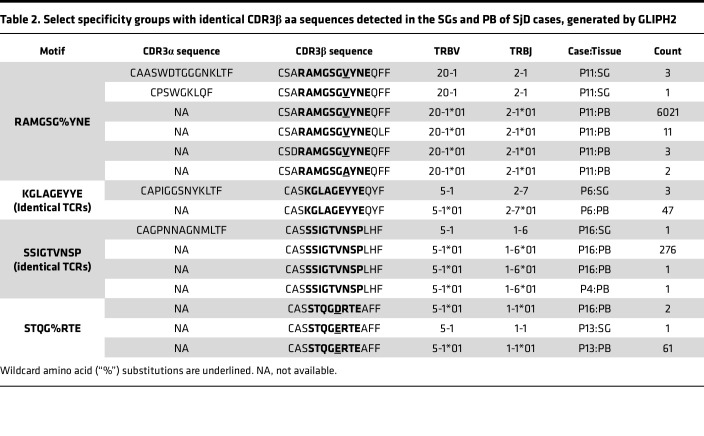
Select specificity groups with identical CDR3β aa sequences detected in the SGs and PB of SjD cases, generated by GLIPH2

**Table 3:**
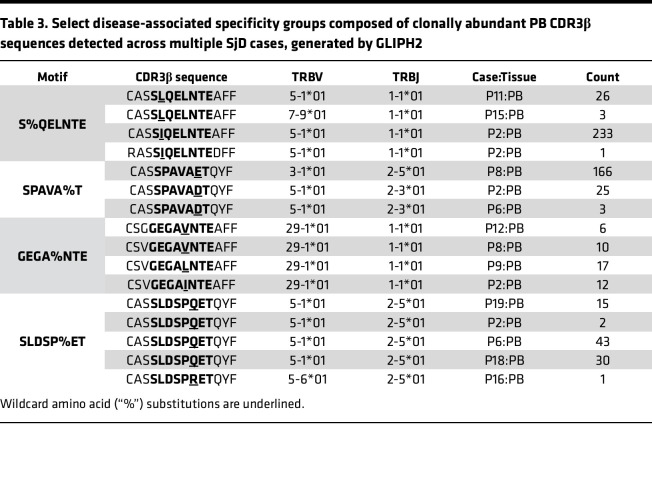
Select disease-associated specificity groups composed of clonally abundant PB CDR3β sequences detected across multiple SjD cases, generated by GLIPH2

## Version 1 12/22/2025

Electronic publication
