# Supplementary material for: Identification of Sjögren’s disease–associated T cell receptor motifs through deep sequencing
Source: JCI Insight. 2025 Dec 22;10(24):e188496. doi: 10.1172/jci.insight.188496 (PMC12890531; doi:10.1172/jci.insight.188496)
Supplement: Supplemental data [file jciinsight-10-188496-s023.pdf]

Supplemental Information

Supplemental Materials and Methods

Supplemental Figures 1 – 9

Supplemental Materials and Methods

TCR deep sequencing

Pooled TCRβ libraries (n=9) from SjD cases and HCs were sequenced on Illumina NovaSeq paired-end 150-bp runs to a median depth of 19.5 million (M) reads (range: 1.9M – 72.5M) and 20.2M reads (3.9M – 63.9M), respectively. For data pre-processing, we used the Molecular Identifier Groups-based Error Correction (MIGEC) (1) v1.2.9 pipeline from https://github.com/mikessh/MIGEC/releases. Pooled libraries were de-multiplexed by extracting sample-specific barcodes and UMI labels from sequences using the *Checkout* routine. Reads with the same UMI were assembled into consensus groups using the *AssembleBatch* routine. UMI-labeling enables MIGEC to track clonality and employ a two-stage filtration system that corrects for PCR amplification biases and removes PCR/sequencing errors while preserving quantitative information even from rare clonotypes. The *CdrBlast* routine was used to extract TCRβ-CDR3 sequences followed by mapping the respective V-D-J gene segment usage, and calculation of clonotype statistics to generate a final CDR3 metadata table.

PB-TCR repertoire statistics

Clonotype statistics and other relevant metrics were calculated and visualized using Immunarch 0.9.0 (2). Calculation of the numbers of unique TCR-encoding cDNAs, CDR3β clonotypes, and clonotype abundance distribution were completed using the *repExplore* function. Relative abundance of clonotypes based on their frequency in the repertoire, a surrogate for degree of clonal expansion in bulk sequencing data, was calculated using the '*.homeo*' option of the *repClonality* function. TRBV and TRBJ gene usage proportions were calculated using the *geneUsage* function. Diversity metrics including Chao1 estimate, and D50 diversity index were calculated using the *repDiversity* function. Rarefaction curves were generated using the *RarefactionPlot* function, and Efron Thisted estimate and Shannon-Weiner index were calculated

using *CalcDiversityStats* function, on VDJtools version 1.2.1 (3). Public TCRs with viral specificities were annotated using VDJdb, McPAS-TCR and PIRD TBAdb databases, through the *dbannotate* function of Immunarch. Comparative analysis between cases and controls was done by grouping data into two groups using the '*.by*' argument in Immunarch, and the '– *factor*' argument in VDJtools.

Generation probability analysis (OLGA)

The pGen values of CDR3β amino acid sequences were calculated from a generative model of VDJ recombination using the OLGA v1.2.4 software (https://github.com/statbiophys/OLGA) (4), available on PyPI (Python package index). Unique CDR3β clonotypes from all SjD cases and controls were used to calculate pGen scores separately for each group. The mean abundances of CDR3β amino acid sequences detected in SjD cases and HCs clearing the sampling threshold of >25,000 unique TCRβ clonotypes (n=17 for both cases and HCs) were calculated for each group respectively. General linear regression was used to model the interaction between pGen and corresponding mean abundances of CDR3β sequences in the two groups through the *glm* (generalized linear model) function on R version 4.1.2 (5). The effect size (β_3) that shows how much the pGen of clonotypes changes with a unit increase in their mean abundance, between case and control repertoires (interaction term), was calculated alongside the corresponding p-values using the following regression model:

$$pGen = \beta 0 + \left(\beta 1 \times mean_{freq}\right) + (\beta 2 \times binary\ variable) + (\beta 3 \times interaction\ term)$$

Here, β_0 is the intercept while β1, β2, and β3 are coefficients associated with change in the mean abundance of CDR3β sequences, relative change in pGen between the two groups, and the interaction between β1 and β2, respectively. The linear fit between pGen and mean abundance was plotted using the ggplot2 package (6). Bootstrapping through *resample* function (R v4.1.2) to assess statistical significance of β3 for randomized subsets of the original deep sequencing data was performed for case and control repertoires individually for a total of 10,000 iterations. The

resulting distribution of β3 and corresponding p values were plotted using the *histogram* function in R.

GLIPH2 TCR-clustering and motif analyses

TCRs were clustered according to the probability that they recognize the same antigen using the GLIPH2 algorithm (7), available at (http://50.255.35.37:8080/tools), implemented on a single input file containing CDR3β sequences obtained from the PB of all cases and controls, and the CDR3 sequences (paired and CDR3β) obtained from the SG of SjD cases. The algorithm was run using the human v2.0 reference files for $CD4^+$ T cells with the following configuration parameters: local_min_pvalue = 0.001, p_depth = 1000, global_convergence_cutoff = 1, simulation_depth = 1000, kmer_min_depth = 3, local_min_OVE = 10, algorithm = GLIPH2, all_aa_interchangeable = 0. Operations on the output cluster file including GLIPH2 score-based filtration, generation of cluster subsets, and calculation of motif-comprising clonotype abundances across all subjects were performed using bash scripts. Statistical comparison of specificity group incidence and clonotype abundances between cases and controls or between PB and SG repertoires of cases, and correlations between motif abundance and clinical disease measures were calculated using GraphPad PRISM 9.5.1.

Cell lines

Engineered 5KC murine hybridoma cell lines were cultured in IMDM (Gibco) supplemented with 10% (v/v) heat-inactivated FBS (Gibco), penicillin-streptomycin at 50 U/mL (Gibco), and 55 μM 2-ME (Sigma). All M12C3 murine B-cell lines used as antigen-presenting cells were cultured in RPMI (Gibco), supplemented with 10% (v/v) heat-inactivated FBS, 1 mM sodium pyruvate (Gibco), 1x MEM NEAA (Gibco), 50 U/mL penicillin-streptomycin and 55 μM 2-ME. Phoenix-Eco cells (ATCC® CRL-3214™) used for packaging retroviruses carrying TCR genes were cultured in DMEM (Gibco), supplemented with 10% (v/v) heat-inactivated FBS, 10 mM HEPES (Gibco), 1

mM sodium pyruvate, 1x MEM NEAA, 50 U/mL penicillin-streptomycin, and 55 μM 2-ME. The EBV-transformed B-lymphoblastoid cell line (B-LCL) was cultured in RPMI (Gibco) supplemented with 10% (v/v) heat-inactivated FBS (Gibco), penicillin-streptomycin at 50 U/mL (Gibco), and 55 μM 2-ME (Sigma). B16-F0 melanoma cells were cultured in DMEM (Sigma) supplemented with 10% (v/v) heat-inactivated FBS (Gibco), penicillin-streptomycin at 50 U/mL (Gibco), and 55 μM 2-ME (Sigma). For the transfection of Phoenix-Eco cells, Phoenix-Eco media lacking antibiotics and FBS was used. CTL-test™ serum-free media (CTL) supplemented with 50 U/mL penicillin-streptomycin (Gibco) was used for re-suspending 5KC and M12C3 cells and co-culturing with antigenic peptides in ELISpot assays.

Peptide libraries

5KC hybridomas expressing SjD-TCRs were screened for reactivity against peptides derived from Ro60 (TROVE2; UniProt Accession ID: P10155-1), Ro52 (TRIM21; P19474-1) and La (SS-B; P05455) using ELISpot assays **(Supplemental Table 12)**. Peptides containing sequences from additional isoforms of Ro60 (P10155-2, P10155-3, P10155-4, P10155-5) and Ro52 (P19474-2) were also tested. Overlapping 15-mer peptides with an offset of four amino acids (consecutive peptides overlapping by 11 amino acids) were synthesized (Pepsets™, Mimotopes), and reconstituted according to the manufacturer's instructions using DMSO (< 0.1% of final concentration), nuclease free water, and aqueous solvents (20 mM $NaHCO_3$ or CH_3COOH) to a final stock concentration of 20-25 mg/mL. Reconstituted peptides were treated with inert gas and frozen for long-term storage at -80°C. Pepsets™ were diluted in CTL-Test™ media (CTL) for all assays. These 15-mers were designed with an overlap of 11 amino acids to ensure that each possible epitope is represented by at least one peptide.

Flow cytometry

TCR-expressing 5KC hybridoma cells were evaluated for surface-expression of TCRβ, CD3 and CD4 on the day of T cell stimulation. Similar evaluation of HLA-DR3, HLA-DQ2, and CD19 was performed on M12C3 APCs. Expression of HLA-DR3 was evaluated on DC-enriched splenocytes and EBV-transformed B-LCLs. 5KC cell lines were stained with the following antibodies: i) BV421-anti-human CD4 (clone OKT4, BioLegend), ii) APC-anti-mouse CD3 (clone 17A2, BioLegend), and iii) PE-Cy7-anti-mouse TCRβ (clone H57-597, BioLegend), while M12C3 APCs were stained with: i) APC-R700-anti-human HLA-DR (clone L243, BD Biosciences), ii) BV421-anti-human HLA-DQ (clone Tu169, BD Biosciences), and iii) PE-Cy7-anti-mouse CD19 (clone 1D3, BD Biosciences). BV510-anti-mouse CD3 (clone 17A2, BioLegend) was used to stain 5KC cell lines used in the ELISpot assays with full-length Ro60. The fluorescence intensity of stained cells was evaluated on a BD FACSCelesta™ and analyzed using FlowJo™ (BD Biosciences).

Measurement of T cell responses to full-length Ro60 protein

Reactivity of 5KC cells expressing the TCR clones 4A and 5B to full-length recombinant Ro60 was measured using the Immunospot® mouse-IL2 single-color ELISpot kit (CTL). The first set of these experiments were conducted by using dendritic cell (DC)-enriched murine splenocytes as APCs. Ro60 protein (Surmodics Inc.) was cleaned of endotoxin using Pierce™ High-Capacity Endotoxin Removal Spin Columns (Thermo Scientific). The protein was then diluted in CTL-test™ serum-free media before adding at half log-fold serial dilutions starting at a final concentration of 10 ug/mL in duplicate wells of an ELISpot plate coated with an IL-2 capture antibody (CTL). To generate DC-enriched splenocytes, an 11-week-old female C57BL/6 HLA-DR3 transgenic mouse, provided by Dr. Ashutosh K. Mangalam (University of Iowa, Iowa City), was injected subcutaneously in the right flank with 15 million Flt3L-transfected B16-F0 melanoma cells, provided by Dr. Susan Kovats (Oklahoma Medical Research Foundation (OMRF), Oklahoma City). Splenocytes harvested 9 days after injection were added (50,000 cells/well) to the ELISpot

plate with and without pre-incubation with purified NA/LE Mouse Anti-Human HLA-DR blocking antibody (Clone L243, BD Biosciences) or purified NA/LE Mouse IgG2a, κ isotype control (Clone G155-178, BD Biosciences) for 30 minutes at 37°C, at a final concentration of 10 µg/mL. Finally, 5KC-4A and 5KC-5B cells (50,000 cells/well) were added and the ELISpot plate was placed in an incubator for 24h at 37°C and 5% CO_2.

5KC-5B cells were additionally screened for reactivity to full-length Ro60 using an EBV-transformed B-lymphoblastoid cell line (B-LCL, provided by the Oklahoma Sjögren's Research clinic, OMRF) autologous to the subject from whom this TCR clone originated. APCs were prepared with and without the pre-incubation of autologous B-LCLs with purified NA/LE Mouse Anti-Human HLA-DR blocking antibody (Clone L243, BD Biosciences) or purified NA/LE Mouse IgG2a, k Isotype Control (Clone G155-178, BD Biosciences) at a final concentration of 10 µg/mL for 30 minutes at 37°C. 5KC-5B cells (50,000 cells/well) and B-LCLs (50,000 cells/well) were mixed with half-log fold serial dilutions of full-length Ro60 protein starting at a final concentration of 10 µg/mL. Here, Ro60 was pre-incubated with and without an equal concentration of autologous IgG isolated from the patient's plasma using the NAb™ Protein A/G Spin Kits (Thermo Scientific), for 30 minutes at 37°C. The ELISpot plate was then incubated for 24h at 37°C and 5% CO_2.

After co-culture, both ELISpot plates were washed and developed using the reagents and protocol provided in the Immunospot™ kit (CTL). IL-2 production was measured by spot-forming units (SFU) on ELISpot plates using an Immunospot™ analyzer (CTL). The mean of IL-2 on duplicate wells was calculated and a threshold for positive responses was set at >2 standard deviations from the mean of IL-2 SFU on negative control (no protein) wells. Stimulation of TCR-expressing 5KC cells with hamster anti-mouse CD3ε antibody (clone 145-2C11, BD Biosciences) at 0.5 µg/mL (in duplicate) was used as a positive control.

References

1. Shugay M, Britanova OV, Merzlyak EM, Turchaninova MA, Mamedov IZ, Tuganbaev TR, et al. Towards error-free profiling of immune repertoires. *Nat Methods.* 2014;11(6):653-5.
2. Nazarov VI, Tsvetkov VO, Fiadziushchanka S, Rumynskiy E, Popov AA, Balashov I, et al.; 2023.
3. Shugay M, Bagaev DV, Turchaninova MA, Bolotin DA, Britanova OV, Putintseva EV, et al. VDJtools: Unifying Post-analysis of T Cell Receptor Repertoires. *PLoS Comput Biol.* 2015;11(11):e1004503.
4. Sethna Z, Elhanati Y, Callan CG, Walczak AM, and Mora T. OLGA: fast computation of generation probabilities of B- and T-cell receptor amino acid sequences and motifs. *Bioinformatics.* 2019;35(17):2974-81.
5. Team RC. Vienna, Austria: R Foundation for Statistical Computing; 2021.
6. Wickham H. *ggplot2: Elegant Graphics for Data Analysis.* Springer-Verlag New York; 2009.
7. Huang H, Wang C, Rubelt F, Scriba TJ, and Davis MM. Analyzing the Mycobacterium tuberculosis immune response by T-cell receptor clustering with GLIPH2 and genome-wide antigen screening. *Nat Biotechnol.* 2020;38(10):1194-202.

Supplemental Figures

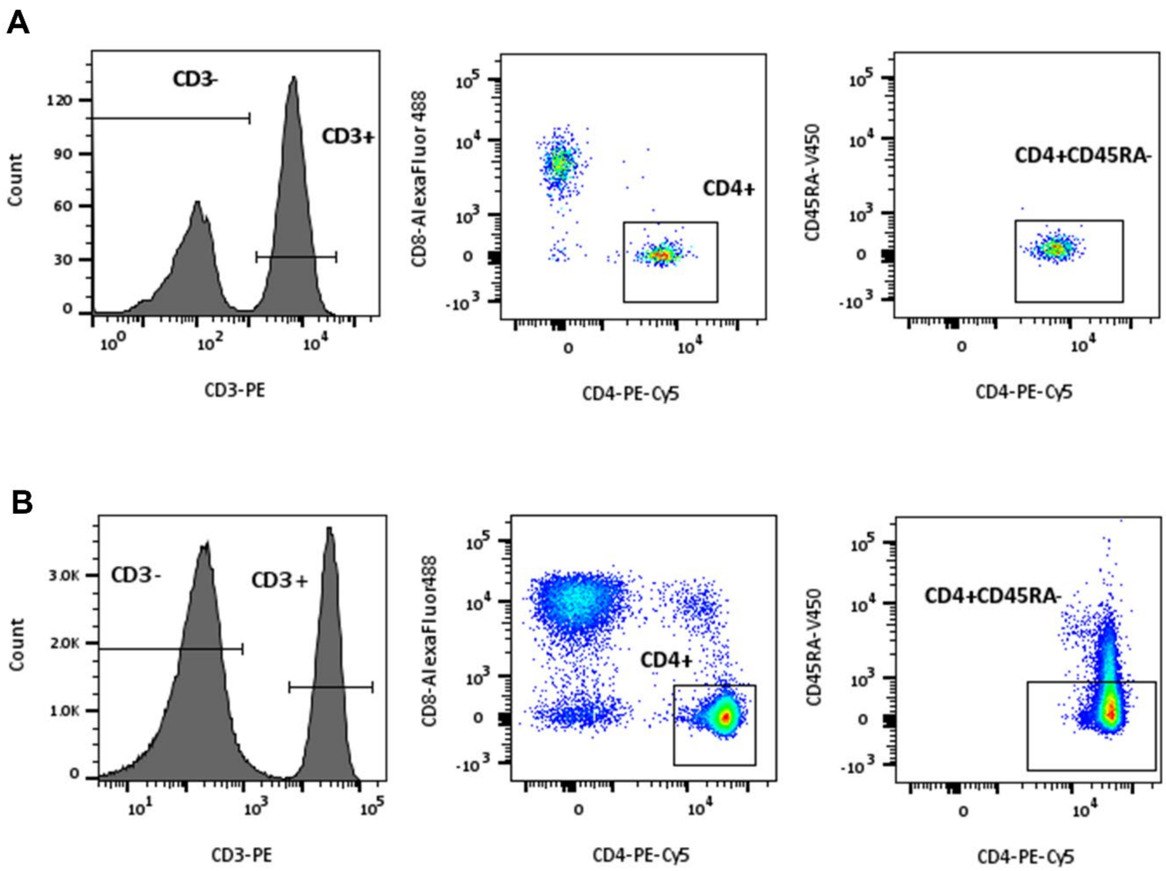

Supplemental Figure 1. Gating strategy for sorting antigen-experienced $CD3^{+}CD4^{+}CD45RA^{—}$ memory T cells. **A)** Single-sorted from biopsied SG tissue of SjD cases. **B)** Bulk-sorted from PBMCs of cases and HCs.

A

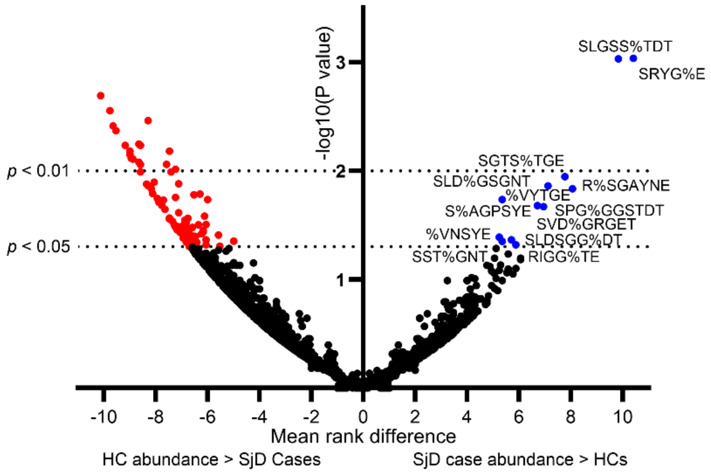

B

V (N) D (N) J

S%WAGRPTDT

Subject :Tissue	CDR3 sequence	Count
	C A S S Y W A G R P T D T Q Y F	
P11 : SG	TGT GCC AGC AGT TAC TGG GCG GGG CGA CCG ACA GAT ACG CAG TAT TTT	4
P11 : PB	TGT GCC AGC AGT TAC TGG GCG GGG CGA CCG ACA GAT ACG CAG TAT TTT	6455
P11 : PB	TGT GCC AGC AGT TAC TGG GCG GGT CGA CCG ACA GAT ACG CAG TAT TTT	2

KGLAGEYYE

Subject :Tissue	CDR3 sequence	Count
	C A S K G L A G E Y Y E Q Y F	
P6 : SG	TGC GCC AGT AAG GGG CTA GCG GGA GAA TAC TAC GAG CAG TAC TTC	3
P6 : PB	TGC GCC AGT AAG GGG CTA GCG GGA GAA TAC TAC GAG CAG TAC TTC	47
P6 : PB	TGC GCC AGT AAG GGG CTA GCG GGA GAA TAT TAC GAG CAG TAC TTC	2

C

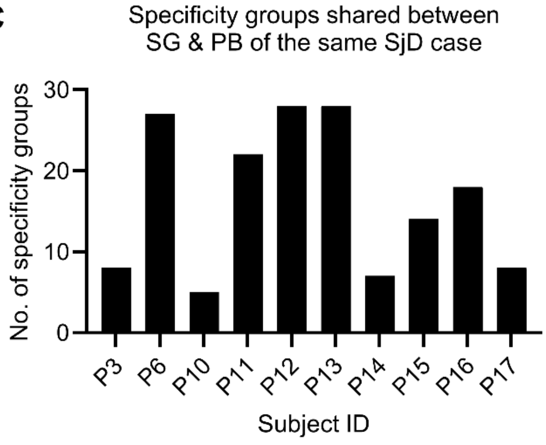

Supplemental Figure 2. Antigen-specificity based clustering analysis reveals trends in TCRβ motif-sharing between SG and PB in SjD cases and HCs. A) Volcano plot comparing the PB abundances of CDR3β sequences constituting SG-PB shared specificity groups (n = 2,634), between SjD cases (n = 17) and HCs (n = 17). TCR motifs significantly enriched among cases (blue) and HCs (red) were determined by *p* value (negative $\log_{10}$ converted *p* values on y axis) and mean rank difference (x axis) from a two-sided Mann Whitney U test. **B)** Select GLIPH2 motifs show clonally expanded SG-T cells expressing identical CDR3β nucleotide sequences that are over-represented in the PB repertoire of the same SjD subject. Color scheme represents V-D-J regions of the TCR-CDR3β sequence, and non-templated nucleotides (highlighted in purple). Underlined nucleotides represent dissimilarity in nucleotide usage at the given position in motif. **C)** Specificity groups comprising TCRs detected in the SG and PB of the same individual are detected in all cases with adequate sampling of both tissues (n = 10). **A) and C),** Adequate sampling thresholds for PB: >25,000 unique clonotypes per subject; SG: >40 TCR+ cells per subject.

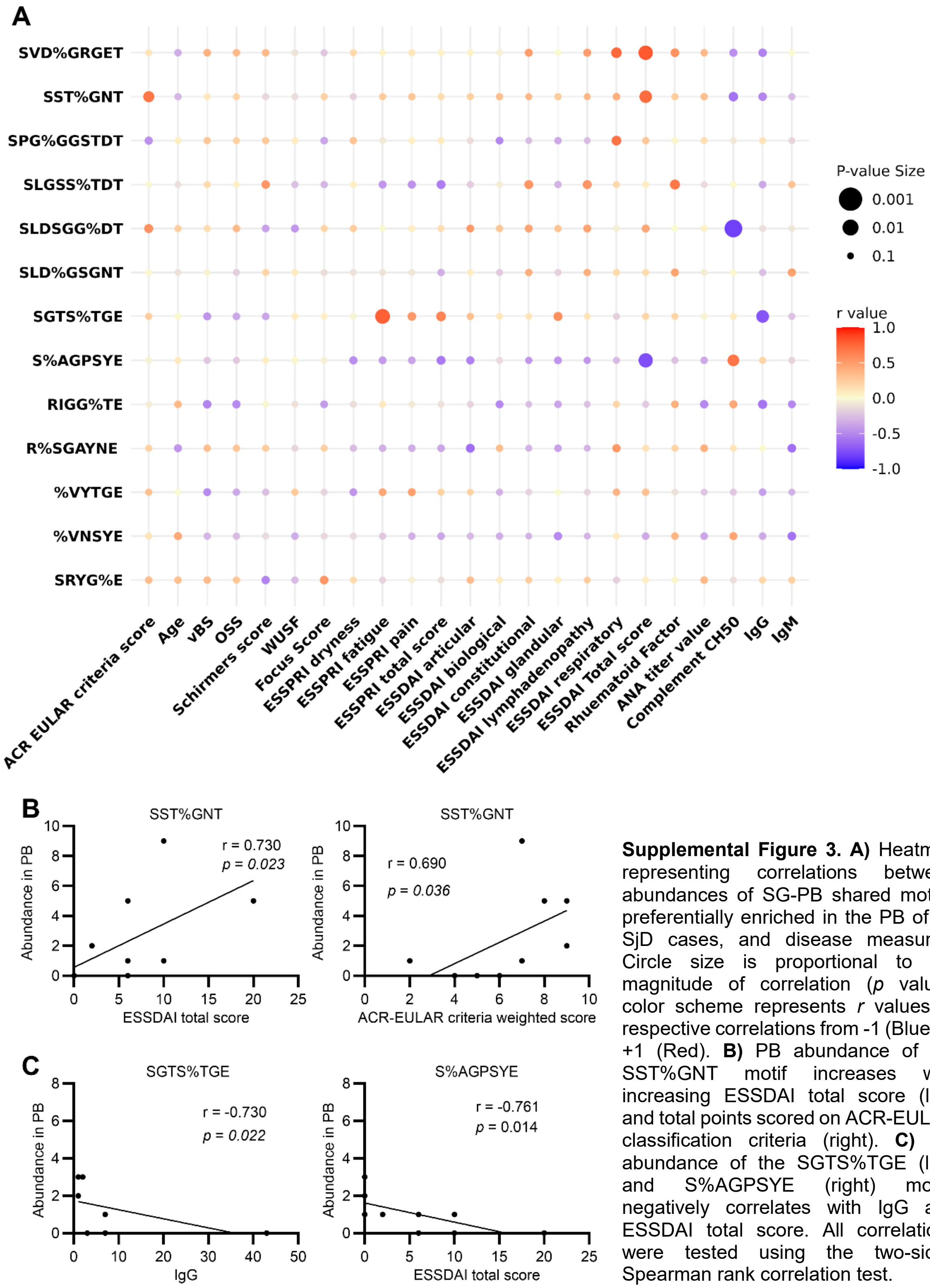

Supplemental Figure 3. A) Heatmap representing correlations between abundances of SG-PB shared motifs, preferentially enriched in the PB of ≥5 SjD cases, and disease measures. Circle size is proportional to the magnitude of correlation (*p* value), color scheme represents *r* values of respective correlations from -1 (Blue) to +1 (Red). **B)** PB abundance of the SST%GNT motif increases with increasing ESSDAI total score (left) and total points scored on ACR-EULAR classification criteria (right). **C)** PB abundance of the SGTS%TGE (left) and S%AGPSYE (right) motifs negatively correlates with IgG and ESSDAI total score. All correlations were tested using the two-sided Spearman rank correlation test.

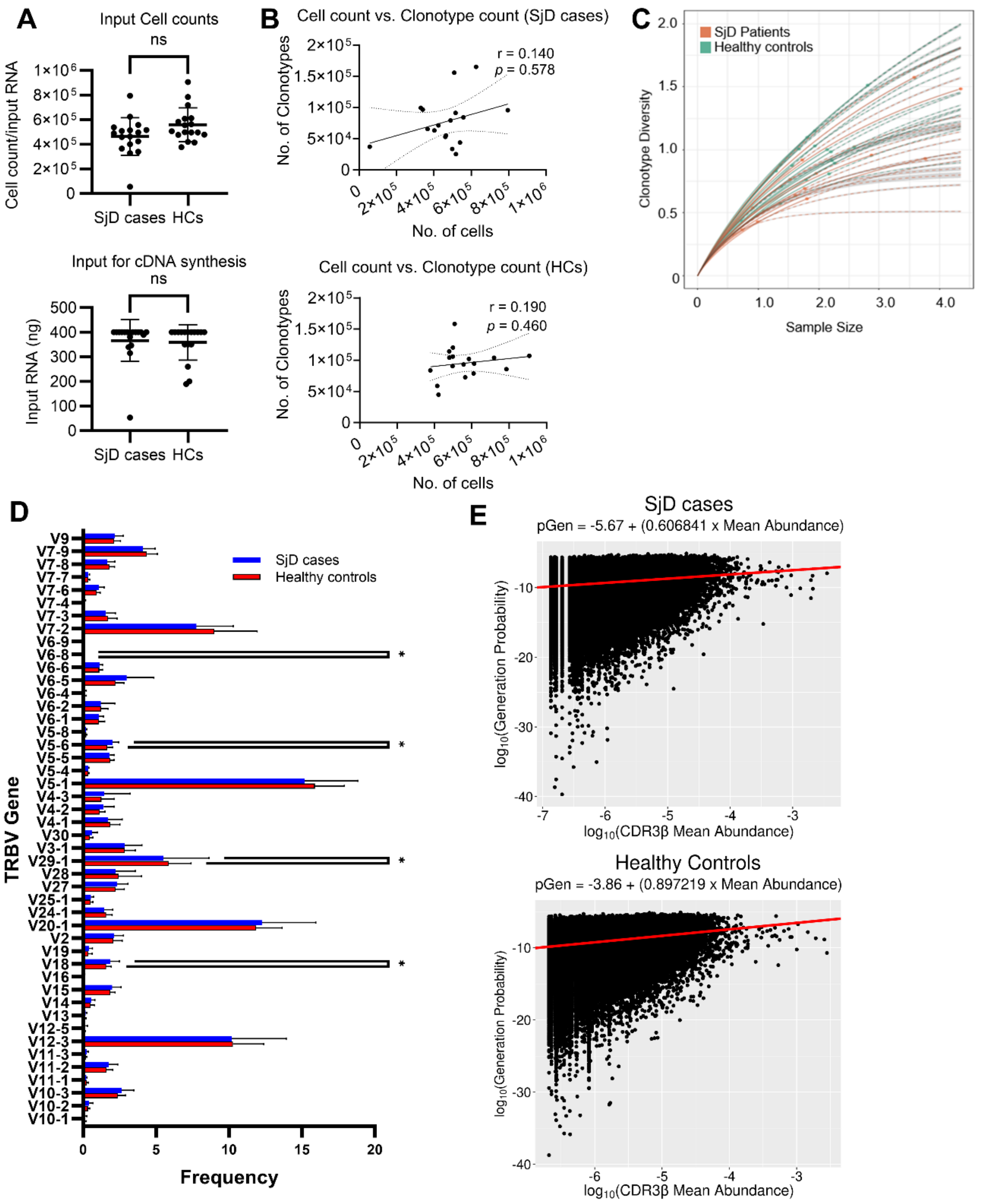

Supplemental Figure 4. PB-TCR repertoire analysis highlights differences between SjD and HC repertoires. A) Number of cells (top) and the corresponding normalized amount of RNA (bottom) used in TCRβ library preparation from SjD cases and HCs; data shown as mean ± SD. **B)** Correlation between number of cells (corresponding to input RNA) used in library preparation in cases and HCs and the corresponding number of CDR3β clonotypes detected in the respective subjects. **C)** Rarefaction curves showing the trend of PB-TCR clonotype diversity (in multiples of 10^5) in cases (orange) and HCs (green) with increasing sample size (no. of TCR-encoding cDNA; in multiples of 10^5). Curves are interpolated from 0 to the original sample size (marked by dots) of each subject and extrapolated up to the largest sample size detected among all subjects. Interpolated and extrapolated regions are shown in solid and dotted lines, respectively, and shaded areas (grey) mark 95% confidence intervals. **D)** TCRβ variable chain gene (TRBV) usage in SjD cases (blue bars) and HCs (red bars). **E)** Scatter plot shows the relationship between mean abundance (x axis) and generation probability (y axis) of PB-CDR3β sequences detected in SjD (top) and HC repertoires (bottom). Axes are $\log_{10}$ converted for plotting and red line shows the slope of linear regression between x and y variables. All comparisons were calculated for cases (n = 17) and HCs (n = 17) with adequate PB clonotype sampling (>25,000 unique clonotypes). **(A)** 2-tailed Welch's t test (top), 2-tailed Mann Whitney U test (bottom), **(B)** 2-tailed Spearman rank correlation test, **(D)** 2-tailed Mann Whitney U test *$p < 0.05$, ns – not significant.

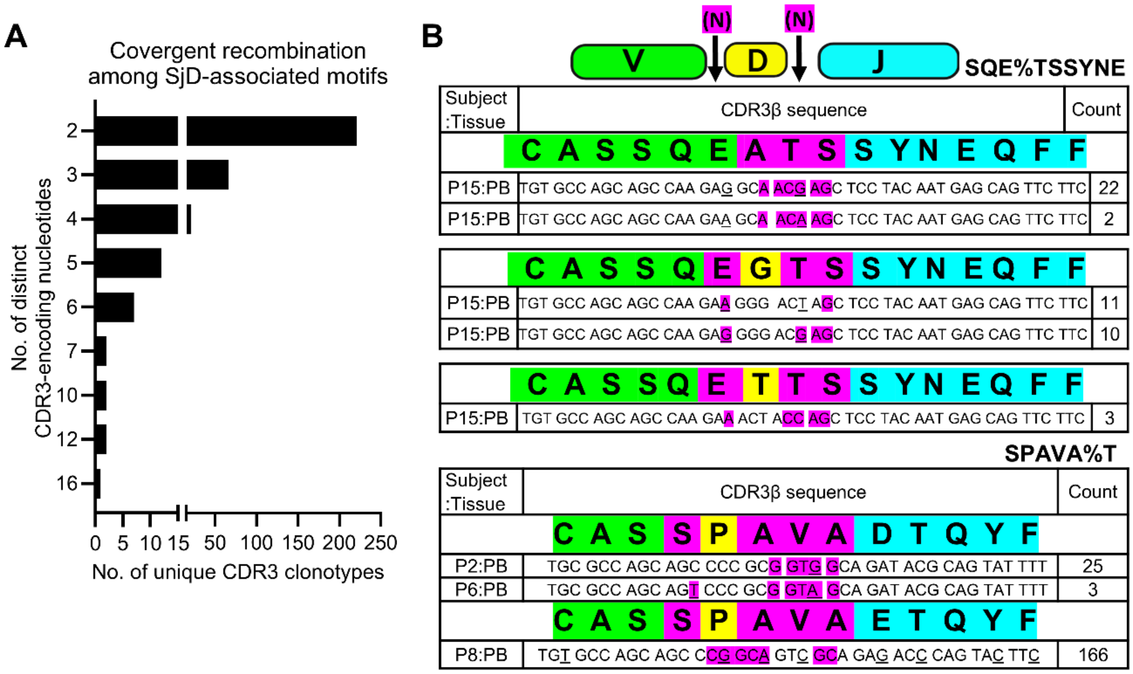

Supplemental Figure 5. Antigen-specificity based clustering analysis reveals disease-associated TCRβ motifs shared between SjD cases. A) Distribution of unique CDR3β amino acid sequences (x axis) constituting disease-associated motifs enriched in SjD cases that are encoded by ≥2 distinct nucleotide sequences (y axis) due to convergent recombination. **B)** TCR antigen-specificity groups reveal clonally abundant CDR3β amino acid sequences exhibiting convergent recombination, both within (SQE%TSSYNE) and across (SPAVA%T) the PB repertoire of SjD cases. Color scheme represents V-D-J regions of the TCR-CDR3β sequences including non-templated nucleotides (highlighted in purple). Underlined nucleotides within a cluster represent dissimilarity in nucleotide usage at the given position.

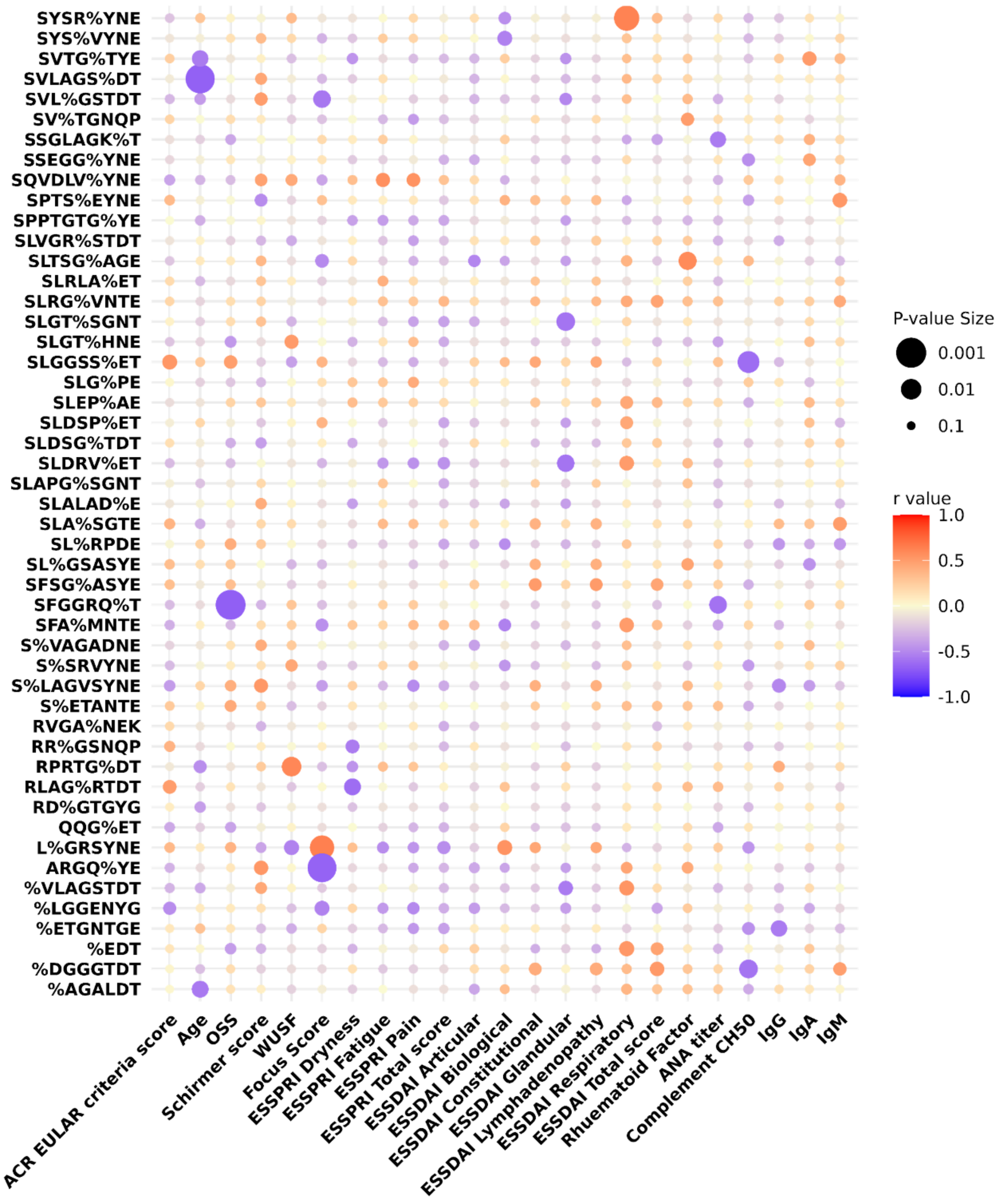

Supplemental Figure 6. Heatmap representing correlations between abundances of disease-associated PB motifs shared among ≥5 SjD cases and disease measures (Two-sided Spearman rank correlation test). Circle size is proportional to the magnitude of correlation (*p* value), color scheme represents *r* values of respective correlations (-1 (Blue) to +1 (Red)).

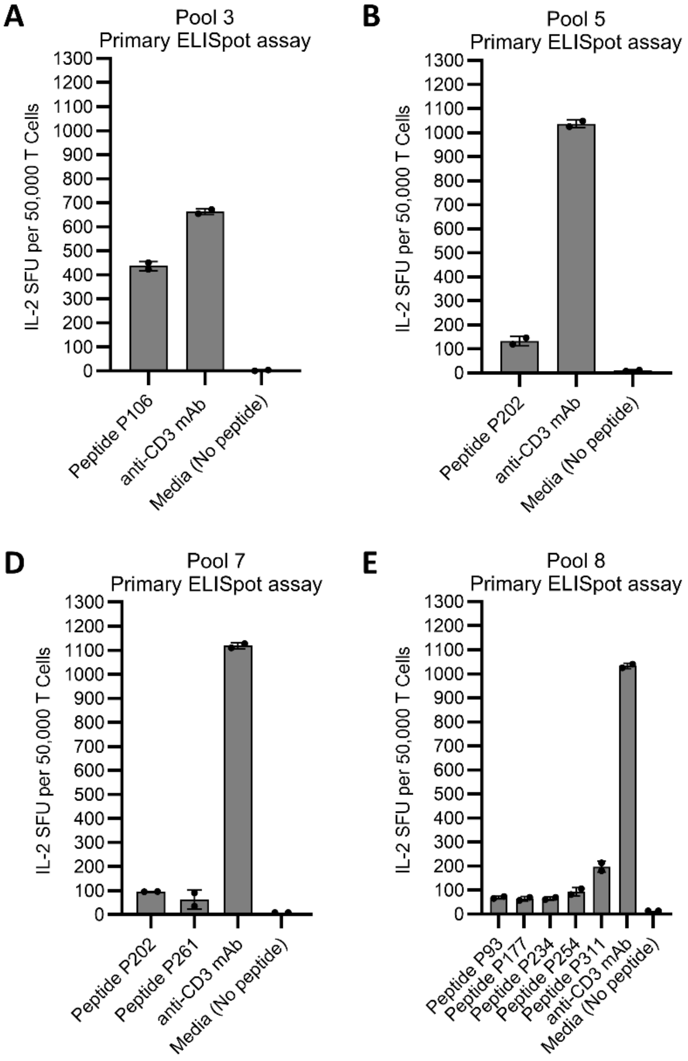

Supplemental Figure 7. TCR transductant pools clearing the threshold for a positive T cell reaction in response to overlapping Ro and La 15-mers. A-E) Bar graphs showing the mean value of IL-2 spot forming units (SFU) generated by 5KC T cell transductants constituting Pool 3 (**A**) Pool 5 (**B**) Pool 6 (**C**) Pool 7 (**D**) and Pool 8 (**E**) on duplicate wells of an ELISpot plate after 24h of incubation with Ro & La overlapping peptides (50 µg/mL), no peptides i.e. media (negative control), and 0.5 µg/mL anti-mouse CD3ε antibody (positive control) in the presence of an equal mixture of HLA-DR3 and HLA-DQ2 transfected APCs; SFU data shown as mean ± SD. The intra-duplicate percentage coefficient of variation (%CV) ranged between 0.93% to 17.2% (Median: 9.4%).

HLA DRB1*03:01 motif (Malcherek, G, et al. (1993) Int Immunol. 5:1229-1237)

```
                      1 2 3 4 5 6 7 8 9
                      L     D   K   L Y
                      I         R     L
                      F         E     F
                      M         Q
                      V         N
Ro 60, P7         W Q V T D M N R L H R F L C F
Ro60, P106    P C P C V T T D M T L Q Q V L M
MAP3K4_4    K D L E E I A A E F R L S A P
MAP3K4_5            E I A A E F R L S A P V R D L
```

Supplemental Figure 8: Ro60 (aa 421-435 and aa 25-39) and MAP3K4 (aa 823-837 and aa 827-841) 15-mers eliciting HLA DR3-restricted T-cell responses through ELISpot assays fit the canonical HLA-DR3 binding motif, showing at least two and three anchor residues, respectively.

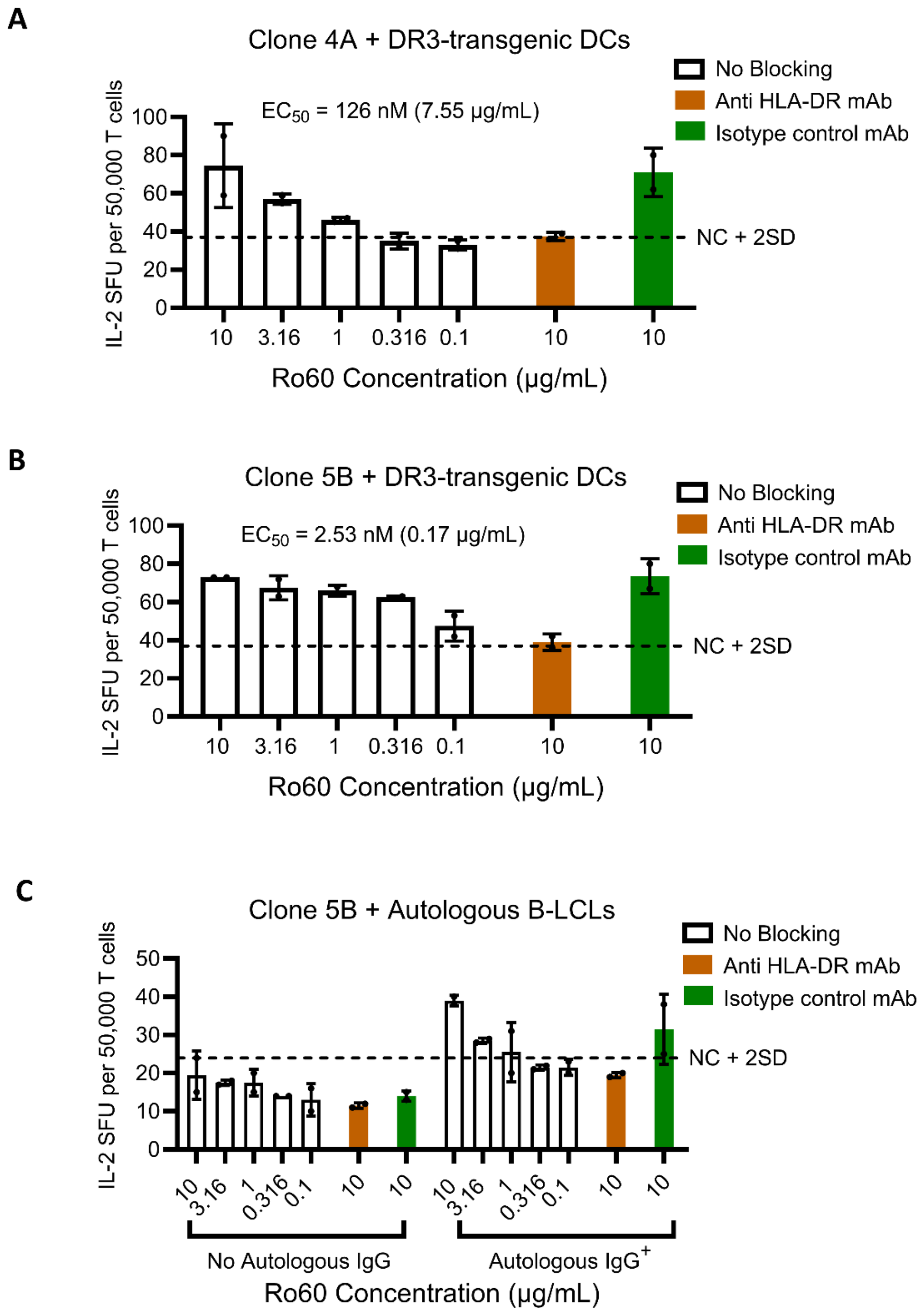

Supplemental Figure 9: TCR transductants generate dose-dependent responses to Ro60 whole protein through natural processing and presentation. A-B) Bar graphs showing the mean value of IL-2 spot forming units (SFU) generated by clones 4A (A) and 5B (B) in response to varying concentrations of full-length recombinant Ro60 protein (white bars) naturally processed and presented by dendritic cell (DC)-enriched splenocytes of a Flt3L-induced HLA-DR3 transgenic C57BL/6 mouse. IL-2 secretion in the presence of anti-HLA-DR mAb (clone L243; brown bars) and the corresponding isotype control mAb (clone G155-178; green bars) is also shown. C) Bar graph shows mean value of IL-2 SFU generated by clone 5B in response to varying concentrations of full-length recombinant Ro60 naturally processed and presented by EBV-transformed B-lymphoblastoid cell lines (B-LCLs) autologous to the subject from whom the TCR clone was derived. IL-2 secretion in the absence and presence of autologous IgG in the reaction well is labeled (white bars), and presence of anti-HLA-DR (brown bars) and isotype control (green bars) is shown. SFU data shown as mean ± SD. Positive response threshold was set at greater than 2SD from mean SFU count on Negative control (NC; no antigen) wells. The intra-duplicate percentage coefficient of variation (%CV) ranged between 0% to 23.1% (Median: 5.7%) (A-B) and 0% to 20.8% (Median: 6.4%) (C).
